# Wheat in vivo RNA structure landscape reveals a prevalent role of RNA structure in modulating translational subgenome expression asymmetry

**DOI:** 10.1186/s13059-021-02549-y

**Published:** 2021-11-30

**Authors:** Xiaofei Yang, Haopeng Yu, Wenqing Sun, Ling Ding, Ji Li, Jitender Cheema, Ricardo Ramirez-Gonzalez, Xuebo Zhao, Azahara C. Martín, Fei Lu, Bao Liu, Cristobal Uauy, Yiliang Ding, Huakun Zhang

**Affiliations:** 1 Key Laboratory of Molecular Epigenetics of the Ministry of Education, Northeast Normal University, Changchun, 130024 China; 2 Department of Cell and Developmental Biology, John Innes Centre, Norwich Research Park, Norwich, NR4 7UH UK; 3 Department of Crop Genetics, John Innes Centre, Norwich Research Park, Norwich, NR4 7UH UK; 4 State Key Laboratory of Plant Cell and Chromosome Engineering, Institute of Genetics and Developmental Biology, The Innovative Academy of Seed Design, Chinese Academy of Sciences, Beijing, China; 5 University of Chinese Academy of Sciences, Beijing, China; 6 CAS-JIC Centre of Excellence for Plant and Microbial Science (CEPAMS), Institute of Genetics and Developmental Biology, Chinese Academy of Sciences, Beijing, China

**Keywords:** Polyploidy, Wheat, Translational regulation, Homoeologous asymmetry, RNA structure, riboSNitches, Xiaofei Yang and Haopeng Yu contributed equally to this study.

## Abstract

**Background:**

Polyploidy, especially allopolyploidy, which entails merging divergent genomes via hybridization and whole-genome duplication (WGD), is a major route to speciation in plants. The duplication among the parental genomes (subgenomes) often leads to one subgenome becoming dominant over the other(s), resulting in subgenome asymmetry in gene content and expression. Polyploid wheats are allopolyploids with most genes present in two (tetraploid) or three (hexaploid) functional copies, which commonly show subgenome expression asymmetry. It is unknown whether a similar subgenome asymmetry exists during translation. We aim to address this key biological question and explore the major contributing factors to subgenome translation asymmetry.

**Results:**

Here, we obtain the first tetraploid wheat translatome and reveal that subgenome expression asymmetry exists at the translational level. We further perform in vivo RNA structure profiling to obtain the wheat RNA structure landscape and find that mRNA structure has a strong impact on translation, independent of GC content. We discover a previously uncharacterized contribution of RNA structure in subgenome translation asymmetry. We identify 3564 single-nucleotide variations (SNVs) across the transcriptomes between the two tetraploid wheat subgenomes, which induce large RNA structure disparities. These SNVs are highly conserved within durum wheat cultivars but are divergent in both domesticated and wild emmer wheat.

**Conclusions:**

We successfully determine both the translatome and in vivo RNA structurome in tetraploid wheat. We reveal that RNA structure serves as an important modulator of translational subgenome expression asymmetry in polyploids. Our work provides a new perspective for molecular breeding of major polyploid crops.

**Supplementary Information:**

The online version contains supplementary material available at 10.1186/s13059-021-02549-y.

## Background

Allopolyploidization (hybridization concomitant with or followed by whole genome duplication) plays an important role in the diversification and speciation of vascular plants [1]. The hybridization and doubling of two or more divergent genomes (subgenomes) in the same nucleus often cause strong and abrupt genetic and epigenetic stresses, resulting in structure and functional incompatibilities [2, 3]. Accumulated evidence indicates that these induced genetic and epigenetic changes in allopolyploids may not only help overcome incompatibilities but also offer a reservoir of novel phenotypes, facilitating their ecological diversification and adaptation to new niches [4, 5]. Of these changes, gene expression subgenome dominance is a common feature of allopolyploids and may have played crucial roles in their adaptation and evolution [6–8]. Wheat represents a textbook example of a recent (~400k years ago) speciation via allopolyploidization and is one of the most successful staple food crops domesticated by humans [9]. Established allopolyploid wheat harbors closely related but distinct subgenomes with meiotic stability and limited intersubgenomic exchange [10]. Moreover, these duplicated genes of polyploid wheat are subject to dynamic selections during domestication and environmental adaptation [11–13].

Recent studies in wheat genomics have focused on the subgenome patterns of transcriptional regulation, revealing asymmetries of RNA abundance in ~30% of homoeologous wheat genes between subgenomes [14]. This transcriptional subgenome asymmetry provides the first step in understanding functional partitioning in wheat. Nevertheless, there is limited knowledge on the translational landscape of wheat subgenomes, which may offer an important perspective in understanding subgenome patterns of gene expression. Translational regulation is one of the most important biological processes that directly controls protein synthesis [15], and it is known from other species that transcriptional regulation of mRNA levels only partially correlates with translation [16]. However, whether translational regulation contributes to subgenome expression patterns in wheat remains elusive.

Over decades, several factors have been identified in regulating translation such as GC content, codon usage, and tRNA copy number, tightly linked with the sequence content [15, 17–21]. RNA secondary structure, as an intrinsic property of RNA molecules, is another important factor contributing to translation regulation [22–25]. Recent technological advances in RNA structure determination, particularly in vivo RNA structure profiling [26–30], has advanced our understanding of mRNA structure and its role in modulating translation. Previous studies in *Arabidopsis* and rice showed that mRNAs with weak structure alongside the strong three-nucleotide periodic pattern in the coding region (CDS) tend to be highly translated, suggesting that mRNA structure may have a general function in modulating translation in plants [26, 31]. Thus, it is of great interest to investigate whether RNA structure features impact on translation in wheat.

The extent of RNA structure folding largely depends on sequence context. The nature of wheat homoeologous genes copies (referred to as homoeologs) provides the perfect opportunity to investigate the impact of single-nucleotide variations (SNVs) on RNA structure where homoeologs from different subgenomes share a certain number of SNVs that fold into similar or different RNA structures within the same cellular environment. The presence of SNVs between wheat subgenomes has supported its extensive adaptation to global environments, through selective breeding by humans over 10,000 years [13, 32]. It is unknown whether these SNVs affect the divergence of RNA structure between wheat homoeologs.

Here, we obtained for the first time, both the translatome and in vivo RNA structurome in wheat. The translatome revealed subgenome asymmetry at the translational level. We found that the single strandedness of mRNA structure, particularly the 5′UTR mRNA structure, is associated with high translation efficiency, suggesting that mRNA structure may have a strong impact on translation in wheat. We discovered that the RNA structure difference between the two subgenomes significantly contributes to translational subgenomes asymmetry where RNA structure plays a prevalent role. We further revealed that the RNA structures of homoeologs sharing more SNVs tend to be more distinct. Subsequently, we identified 3564 SNVs which induced large structure disparities, as riboSNitches between the two subgenomes which are more highly conserved across durum wheat (*Triticum turgidum* ssp. *durum*, BBAA) accessions compared to non-riboSNitches. We found that these riboSNitches were more strongly selected during domestication compared to non-riboSNitches. Further research demonstrated that selected riboSNitches could be shaped through human breeding for their modulation of translation. Based on the above, this study provides a new perspective for wheat genetic research and, more generally, for polyploid crop breeding in the future.

## Results

### Polysome profiling reveals translational subgenome asymmetry in wheat

**Fig. 1:**
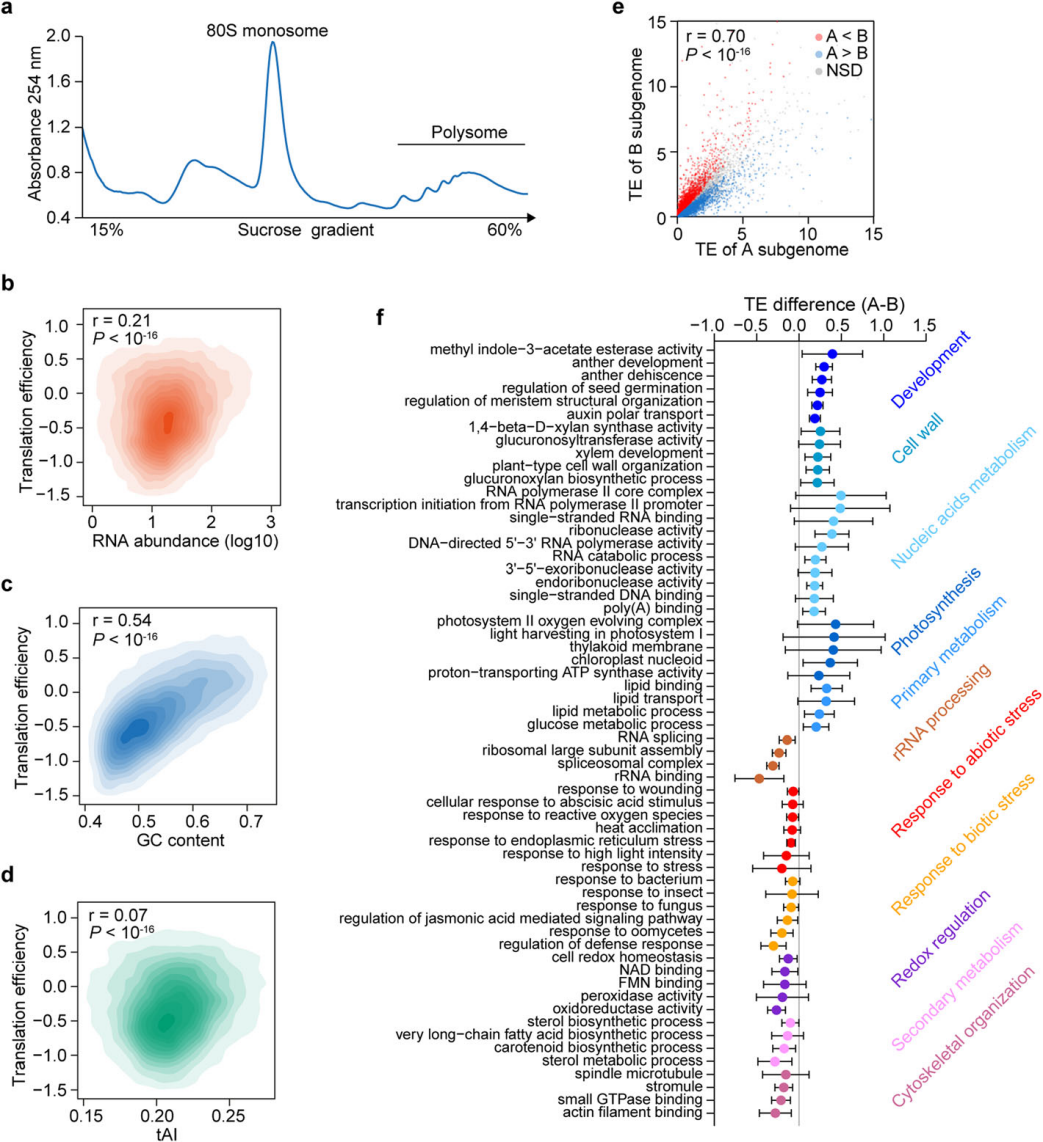
Translational landscape in tetraploid Kronos. **a** Representative chromatogram of Kronos lysates separated by sucrose gradient. The peaks representing 80S monosome and polysome are indicated. **b**–**d** Correlations between translation efficiency (TE) and RNA abundance, GC content, and tRNA adaptation index (tAI) in Kronos. The correlation coefficients between translation efficiency and RNA abundance, GC content, and tAI are 0.21, 0.54, and 0.07, respectively (*P* values < 10^-16^). **e** Scatter plot showing the TE differences between A and B subgenomes. The A subgenome homoeologs with significantly higher or lower TE values than that those in the B subgenome, or without significant differences between A and B subgenomes are coloured with blue, red, or gray, respectively (*P* < 0.05, by Student’s *t* test followed by Benjamini-Hochberg correction, NSD, no significant difference). **f** Translational patterns between the A and B subgenomes of different biological functions. Each listed item includes at least ten homoeologous pairs. The error bar indicates the standard error in TE differences between the homoeologs of the A and B subgenomes

To uncover the translational landscape in wheat, we performed polysome profiling on the tetraploid durum wheat cultivar, Kronos (2*n* = 4*x* = 28, BBAA), by subjecting polysome-associated RNAs to deep sequencing (Fig. 1a, and Additional file 1: Figure S1A, B and C). We then calculated the translation efficiency (TE), the ratio of polysome footprints to mRNA fragments, which represents the level of associated ribosomes on individual mRNA [33]. We found a significant correlation of 0.21 between TE and RNA abundance (Fig. 1b, *r* = 0.21, *P* < 10^-16^), consistent with studies in other species [16]. Thus, our result showed that translational efficiencies of mRNAs in wheat were partially associated with their transcriptional levels.

We then explored the impact of GC content, codon usage, and tRNA copy number on translation. We found that GC content significantly correlated with TE (Fig. 1c, *r* = 0.57, *P* < 10^-16^), indicating mRNAs with high GC content are highly translated in comparison to mRNA with low GC content. Notably, a similar result was also reported in rice [21], suggesting that GC content is likely to be an important factor in modulating translation in plants whose genomes have high GC content. For codon usage, we first measured codon adaptation index (CAI) [17] and found a strong correlation between CAI and TE (*r* = 0.60, *P* < 10^-16^), suggesting codon usage may affect translation (Additional file 1: Figure S2A). We then calculated tRNA adaptation index (tAI), which measures the codon preference and considers the intracellular concentration of tRNA molecules and the efficiencies of each codon–anticodon pairing [18]. Surprisingly, the correlation between tAI and TE was very poor (albeit significant), with a correlation coefficient of 0.07 (Fig. 1d, *r* = 0.07, *P* < 10^-16^), indicating that codon preference is unlikely to be a major modulator of translation in wheat.

Previous studies suggested that transcriptional subgenome asymmetry could potentially represent the first step towards neo- or subfunctionalization of wheat homoeologs [14]. We then compared the TEs of homoeologs between the two subgenomes in tetraploid Kronos. The TEs of A subgenome mRNAs positively correlated with the TEs of their homoeologous B subgenome mRNAs (Fig. 1e, *r* = 0.70, *P* < 10^-16^). Notably, among 13,294 homoeologous pairs with TE values, we found that 7418 gene pairs (~55%) displayed a significant difference in TE values between their A and B subgenomes homoeologs, indicating the existence of translational subgenome asymmetry. We then examined whether the homoeologs with significantly different TEs in the seedling samples examined are associated with specific biological functions. We classified the 7418 genes pairs into individual biological functions and calculated the corresponding averaged TE differences. We found that those homoeologs from the A subgenome with significantly higher TE values than the B subgenome tend to be associated with biological functions related to developmental, cell wall, nucleic acid metabolism, photosynthesis, and primary metabolism (Fig. 1f). While those homoeologs from the A subgenome with significantly lower TE values than the B subgenome tend to be associated with biological functions related to rRNA processing, response to abiotic stress, response to biotic stress, redox regulation, secondary metabolism, and cytoskeletal organization (Fig. 1f).

We then studied which factors contribute to translational subgenome asymmetry. We did not observe significant correlations between TE difference and GC difference nor tAI difference, suggesting that both GC content and codon preference do not significantly contribute to translational subgenome asymmetry (Additional file 1: Figure S2B, C). Altogether, the wheat homoeologous expression asymmetry at the translational level tended to be associated with specific biological functions. However, both GC content and codon preference did not significantly contribute to translational subgenome asymmetry, leading to our subsequent investigation into the role of RNA structure.

### In vivo RNA structure profiling revealed the different RNA structure landscape at the subgenome level of wheat

**Fig. 2:**
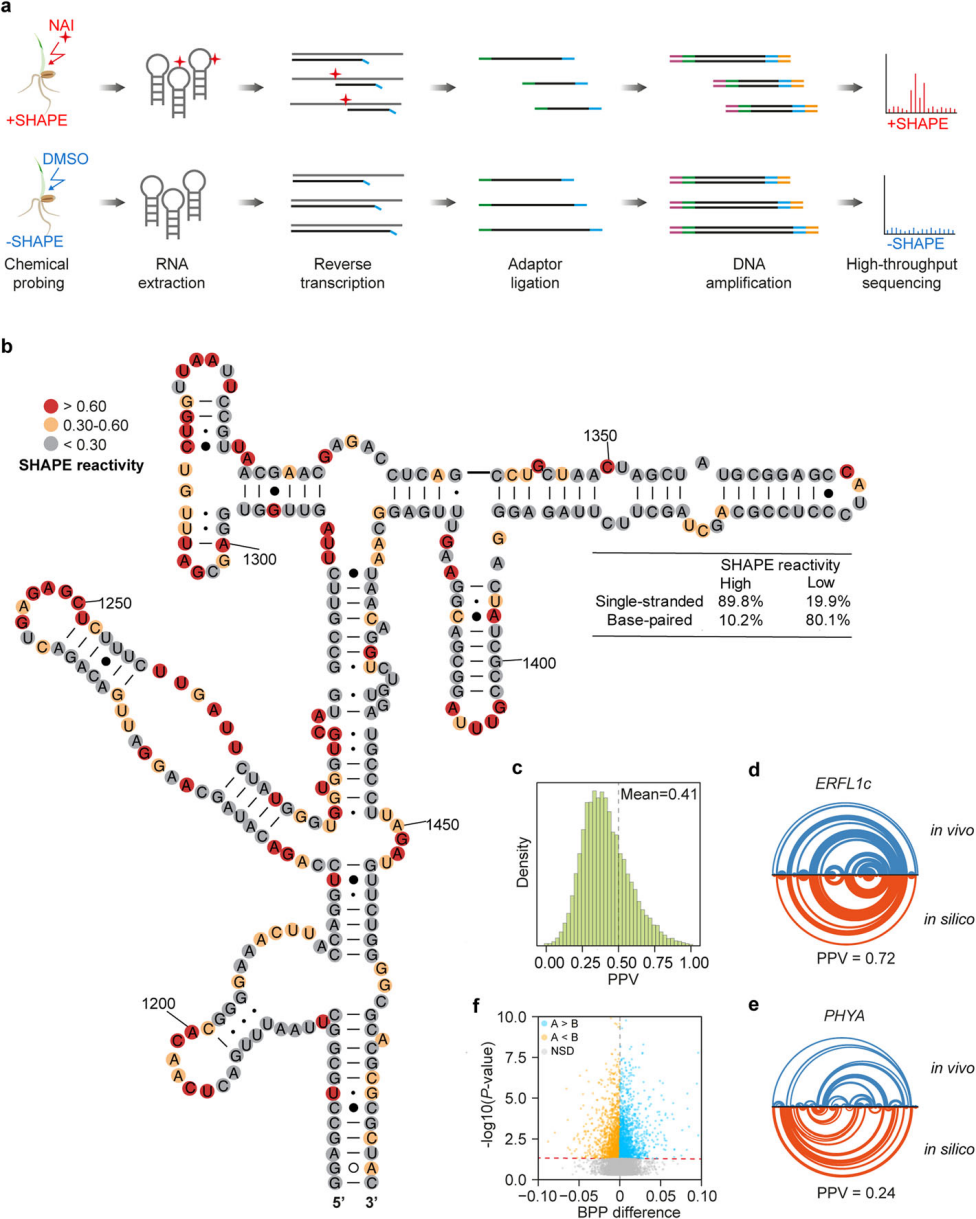
In vivo RNA structure landscape in tetraploid Kronos. **a** Diagram showing the experimental procedures of in vivo RNA structure profiling in wheat. Seedlings were incubated with either NAI (+SHAPE) or DMSO (−SHAPE), respectively. RNA was extracted and subjected to library generation and high-throughput sequencing and analysis. **b** Strong agreement between SHAPE reactivity and phylogenetic structure on 18S rRNA. Nucleotides with SHAPE reactivity lower than 0.3, between 0.3 and 0.6, or over 0.6 are coloured in gray, orange, or red, respectively. A statistical comparison between SHAPE reactivity and base-pairing features in phylogenetic structure is tabulated. **c** Genome-wide comparison of in vivo folded RNA structure and in silico predicted structure. A high or low positive prediction value (PPV) indicates strong or weak agreement between in vivo structure and in silico structure, respectively. **d** Arc diagram showing *ERFL1c* (*ethylene-responsive factor-like transcription factor*, *TRITD3Av1G203860*) transcript with high PPV between in vivo structure and in silico structure, indicating strong agreement between in vivo structure and in silico structure on *ERFL1c*, every arc corresponds to one base pair. **e** Arc diagram showing *PHYA* (*Phytochrome A*, *TRITD4Av1G095120*) transcript with low PPV between in vivo structure and in silico structure, indicating weak agreement between in vivo structure and in silico structure on *PHYA*, otherwise in Fig. 2d. **f** Volcano plot showing differences of the average base-pairing probability (BPP) between the A and B subgenomes. The A subgenome homoeologs with significantly higher or lower average BPP than those in the B subgenome, or without significant differences between the A and B subgenomes are colored in blue, orange, or gray, respectively (*P* < 0.05, by Wilcoxon rank-sum test, NSD, no significant difference)

Our previous studies in both *Arabidopsis* and rice indicated that in vivo RNA structure globally affects translation [26, 31]. Therefore, we determined the in vivo wheat RNA structure landscape by performing SHAPE (**S**elective 2’ **H**ydroxyl **A**cylation analysed by **P**rimer **E**xtension) chemical profiling in the tetraploid durum wheat cultivar Kronos (Fig. 2a). The SHAPE reagent, 2-methylnicotinic acid imidazolide (NAI), probes the single-strandedness of all four RNA nucleotides [34]. We optimized NAI treatment for wheat seedlings and achieved efficient modification using 200 mM NAI treatment for 15 min (Additional file 1: Figure S3A, B). We then generated two independent biological replicates, with and without NAI, following our established Structure-seq library construction pipeline (Fig. 2a) [28]. We generated more than 2.8 billion 150-nt paired-end reads and over 83% reads were uniquely mapped to the transcriptomic reference in durum wheat (Additional file 1: Figure S4A). The Pearson correlation coefficients (PCCs) of mRNA abundances between two biological replicates for both -SHAPE and +SHAPE libraries were very high, indicating high reproducibility (Additional file 1: Figure S4B, C). With high sequencing depths, we were able to achieve nucleotide-resolution coverage for 34,018 transcripts (including 13,294 homoeologous pairs), over half of the wheat expressed transcriptome at seedling stage (Additional file 1: Figure S4D). To validate the accuracy of our SHAPE-Structure-seq libraries, we compared our SHAPE reactivity with phylogenetically derived 18S rRNA structure, which is evolutionarily conserved and is the closest model of in vivo RNA structure of 18S rRNA. We found that our SHAPE-Structure-seq highly agrees with phylogenetically derived 18S rRNA structure (Fig. 2b). We found that 89.8% of nucleotides that showed high in vivo SHAPE reactivity in our data set corresponded to single-stranded regions in the phylogenetic structure (true positive), whereas 80.1% of the nucleotides that showed low in vivo SHAPE reactivity corresponded with base-paired regions in the phylogenetic structure (true negative) (Fig. 2b). These results provide evidence of the high quality and depth of the wheat in vivo RNA structurome.

To further assess RNA structure features, we folded the individual mRNAs with SHAPE reactivities and compared these in vivo RNA structures with in silico RNA structures, which are the most thermostable RNA structures. For each mRNA, we calculated the positive predictive value (PPV) [35], a metric to measure the proportion of base pairs between two structures. A high PPV indicates the in vivo RNA structure is similar to the in silico structure, representing a more thermostable RNA structure. In contrast, a low PPV indicates that the in vivo RNA structure is distinct from the in silico structure, representing a less thermostable structure. We found that most wheat mRNAs did not fold in vivo according to in silico structure, as evidenced by the broad PPV distribution with an average PPV of 0.41 (Fig. 2c). The PPV of wheat RNA structurome is similar to that in *Arabidopsis* (0.38) and lower than that in rice (0.54) [26, 31], suggesting the in vivo RNA structures in wheat maintained their flexibility for folding. We used arc diagrams, a tool for visualizing and comparing base pairs between two RNA structures simultaneously [36], to depict RNA secondary structures both in vivo and in silico. Figure 2d, e illustrate mRNAs of *ERFL1c* (*ethylene-responsive factor-like transcription factor*, *TRITD3Av1G203860*) and *PHYA* (*Phytochrome A*, *TRITD4Av1G095120*) with high PPV (i.e., similar in vivo structure) or low PPV (i.e., distinct in vivo structure), respectively, compared to the predicted in silico structure.

We then compared mRNA structures between the A and B subgenome homoeologs. Due to different sequence lengths between some pairs of homoeologs, we calculated the base-pairing probability (BPP) for each nucleotide of homoeologs derived from in vivo RNA structures, which measures the likelihood of single-strandedness. We then compared the average BPP between homoeologs and found that 20.1% of homoeologs showed significantly higher average BPP in the A subgenome than that in the B subgenome, while 19.4% of homoeologous pairs showed significantly lower average BPP in the A subgenome compared to the B subgenome (Fig. 2f, Additional file 3: Table S2). Therefore, the RNA structures for 39.5% of tetraploid homoeologous pairs vary significantly from each other between the A and B subgenomes.

### RNA structure is an important modulator of translation in wheat

**Fig. 3:**
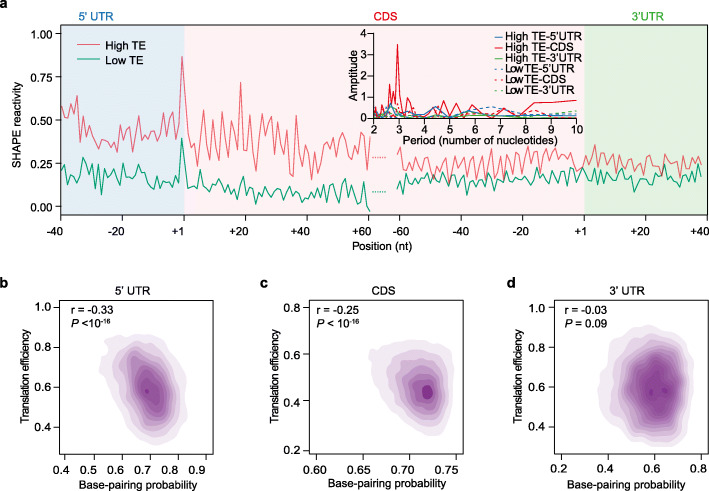
Translation efficiency (TE) correlates with in vivo RNA structures in tetraploid Kronos*.* **a** Line plot showing SHAPE reactivities of highly and lowly translated transcripts, as shown by 10% of highest or lowest TEs. SHAPE reactivities of different genic regions across highly (red) or lowly translated (bluish green) mRNAs were averaged. mRNAs were aligned by their translation start and stop codons. The inset shows strong 3nt periodicity of SHAPE reactivity in the CDS region, but not in the 5′UTR or 3′UTR of highly translated mRNAs, nor in the 5′UTR, CDS, or 3′UTR of lowly translated mRNAs. **b–d** Relationship between RNA structure and TE across different genic regions. The scatter plots showed the correlation between base-pairing probability and TE across 5′UTR (**b**), CDS (**c**), and 3′UTR (**d**)

Having generated the in vivo RNA structurome in tetraploid Kronos, we then investigated whether RNA structures influence translation. We compared the average SHAPE reactivities between mRNAs with the highest 10% TEs and the lowest 10% TEs. We found that mRNAs with the highest TEs showed significantly higher SHAPE reactivities than those mRNAs with the lowest TEs across both UTR and CDS regions (Fig. 3a). We then examined the 3-nt periodicity, an RNA structure feature indicative of high translation [26, 37, 38]. The SHAPE reactivity profile showed a significantly higher magnitude of 3-nt periodicity in the CDS of mRNAs with highest TEs, compared to that for the UTRs of these mRNAs, and that in all genic regions of mRNAs with the lowest TEs (Fig. 3a, inset). We found a significant anti-correlation between average BPP and TE in both 5′UTR and CDS regions, with correlation coefficients of −0.33 and −0.25, respectively (Fig. 3b, c, *P* values < 10^-16^), indicating mRNAs with weaker structures in the 5′UTR or CDS regions tend to be highly translated (Fig. 3b, c). RNA structures in 3′UTR did not correlate with the TEs, suggesting 3′UTR structures do not significantly affect translation (Fig. 3d).

Previous studies on both in silico and in vitro RNA structures suggested that structured RNAs tend to have a high GC content [39]. Since GC content was strongly associated with translation efficiency (Fig. 1c), we then investigated whether GC content affects translation via its impact on the RNA structure. We correlated average BPP with GC content and found that GC content did not strongly correlate with in vivo RNA structures at both 5′UTR and CDS regions, but was more strongly associated with 3′UTR mRNA structure (Additional file 1: Figure S5, with r values of 0.05, −0.02, and 0.31 for 5′UTR, CDS, and 3′UTR, respectively). Thus, these results show that the correlations between RNA structure and translation in both 5′UTR and CDS are unlikely to be associated with the impact of GC content on translation. Therefore, it is likely that both RNA structure and GC content independently serve as major modulators of translation in wheat.

### RNA structure plays a prevalent role in translational subgenome asymmetry

**Fig. 4:**
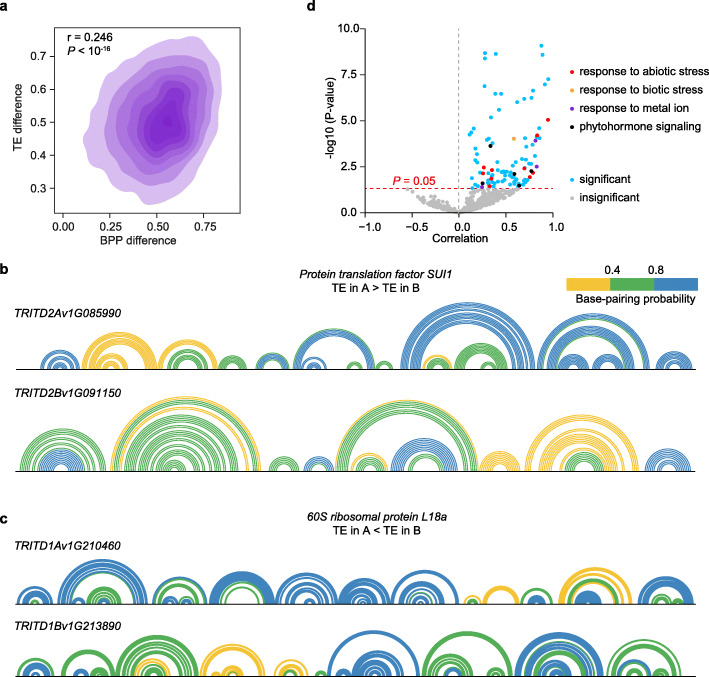
RNA structure contributes to translational subgenome asymmetry in tetraploid Kronos. **a** Scatter plot showing the correlation between the absolute values of difference of average base-pairing probability (BPP) and translation efficiency (TE) difference between the A and B subgenome homoeologs (*n* = 4283, *P* < 10^-16^). **b** Arc diagram showing the RNA structure of the homoeologous pair of *protein translation factor SUI1*, *TRITD2Av1G085990* in the A subgenome, and *TRITD2Bv1G091150* in the B subgenome, with higher TE in the A subgenome than in the B subgenome. Every arc corresponds to one base pair, the pairing nucleotides with pairing probability lower than 0.4, between 0.4 and 0.8, or over 0.8.are indicated by lines in yellow, green, or blue, respectively. **c** Arc diagram showing the RNA structure of the homoeologous pair of *60S ribosomal protein L18a*, *TRITD1Av1G210460* in the A subgenome, and *TRITD1Bv1G213890* in the B subgenome, with lower translation efficiency in the A subgenome than that in the B subgenome. Otherwise in Fig. 4b. **d** Volcano plot showing the correlation coefficients between difference of average BPP and TE difference, for genes related to different GO functions. The red line indicates the cut-off *P value* of 0.05, the points above the red line indicate a significant correlation between the BPP difference and TE difference for the groups of homoeologs with specific functions. The GO function items related to abiotic stress response, biotic stress response, metal ion response, phytohormone signaling are colored in red, orange, purple, or black, respectively

Following our discovery of the impact of RNA structure on translation in wheat, we further asked whether RNA structure influences translational subgenome asymmetry. We assessed the correlation between differences of average BPP and TE differences between homoeologs. We found that differences of average BPP for the full-length mRNA positively correlated with TE differences, whereby the bigger the difference of RNA structure between homoeologs, the greater the difference of TEs (Fig. 4a, *r* = 0.25, *P* < 10^-16^). Among different genic regions, the association between RNA structure difference and TE difference was stronger in both 5′UTR and CDS while moderately in 3′UTR (0.29, 0.27, and 0.11, *P* values < 0.001). To illustrate this association between RNA structure difference and TE difference, we plotted in vivo mRNA structure models highlighted with average BPP for these two factors. *Protein translation factor SUI1* with a significantly higher TE in the A subgenome than in the B subgenome also had greater single-strandedness in its A subgenome RNA structure and lower BPPs and higher SHAPE reactivities, compared to the B subgenome (Fig. 4b and Additional file 1: Figure S7A). In contrast, *60S ribosomal protein L18a* with A subgenome TE values significantly lower than those in the B subgenome had less single-strandedness in its A subgenome RNA structure with higher BPPs and lower SHAPE reactivities, compared to the B subgenome (Fig. 4c and Additional file 1: Figure S7C). Additional examples are presented in Additional file 1 Figures S6A-B, S7B and S7D. Of note, the link between high TE and weak RNA structure is more obvious in 5′UTR and less obvious in 3′UTR, emphasizing a predominant role of RNA structure in 5′UTR affecting translation efficiency.

To assess the biological functions of those homoeologs showing a strong association between RNA structure difference and TE difference, we grouped the homoeologs based on their biological functions and assessed the correlation between difference of average BPP and TE difference for each biological function group. We found that the homoeologs with biological functions related to abiotic stress response, biotic stress response, metal ion response, and phytohormone signalling display significantly higher correlations between RNA structure difference and TE difference (Fig. 4d), implying a close link between RNA structure and translation efficiency in these homoeologs. Taken together, our results indicated that RNA structure has a more prevalent role in modulating translational subgenome asymmetry, compared to GC content and codon preference.

### Impact of single-nucleotide variations (SNVs) between homoeologs on RNA structure

**Fig. 5:**
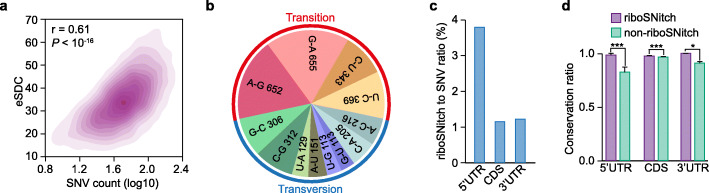
RiboSNitches landscape in tetraploid Kronos. **a** Scatter plot showing the correlation between experimental structure disruption coefficient (eSDC) and single-nucleotide variations (SNV) between A and B subgenome. **b** Statistics of nucleotide transition or transversion for riboSNitches. **c** Bar plot showing the ratio of riboSNitches relative to the SNVs at different genic regions. **d** Comparison of conservation ratio between riboSNitch SNVs and non-riboSNitch SNVs at different genic regions, data collected from 13 accessions of tetraploid *T. turgidum* spp. *durum* [13], error bar indicates se (*P* values, 0.002, 0.005, and 0.017 for 5′UTR, CDS and 3′UTR, respectively, by Student’s *t* test).

Single-nucleotide variation (SNV) has a strongly impact in affecting RNA structure folding [40]. We found a ratio of 4.58% for SNV between A and B subgenomes, which led us to explore the impact of these SNVs on the structures of homoeologs. We evaluated the disruptive effect of a SNP on an RNA structure by calculating the experimental structure disruption coefficient (eSDC) between two homoeologs [27, 41]. We found a strong correlation between the numbers of SNVs and eSDC (Fig. 5a, *r* = 0.61, *P* < 10^-16^), implying that the greater the number of SNVs between homoeologs led to greater differences in RNA structure. Conversely, the correlation between the length of inserted/deleted nucleotides and eSDC between subgenomes were low (*r* = 0.12 and *r* = 0.10 for A and B subgenomes, respectively), suggesting a weak impact of nucleotide insertion/deletion in differentiating the RNA structure of homoeologs (Additional file 1: Figure S8A, B). Subsequently, out of 304,835 SNVs, we identified 3564 SNVs (ratio = 1.17%) inducing large RNA structure disparities, termed “riboSNitches” (Additional file 1: Figure S8C, D and Additional file 4: Table S3, Additional file 5: Table S4) [27, 42]. Among these riboSNitches, the amount of transition (i.e., similar shape base interchanges) riboSNitches was slightly larger than that of transversion (i.e., dissimilar shape base interchanges) riboSNitches (Fig. 5b, Additional file 1: Figure S8C, D), with A-G and/or G-A transition highly enriched (Fig. 5b). Among different genic regions, we found that the ratio of riboSNitches in 5′UTR was over three times higher than those in CDS and 3′UTR regions (Fig. 5c).

Since riboSNitches tend to be conserved due to their notable impact on RNA structures and corresponding functions [40], we then asked whether these riboSNitches tend to be conserved across tetraploid *T. turgidum* spp. *durum*. We found that riboSNitches in different genic regions were more conserved than non-riboSNitches (i.e., SNVs that did not cause significant RNA structure changes; Fig. 5d). In particular, riboSNitches in 5′UTR were more highly conserved with 98.4% compared to 82.4% for non-riboSNitches (Fig. 5d). Therefore, our results suggest that SNVs significantly impact on RNA structure differences between homoeologs and those riboSNitches tend to be highly conserved across tetraploid *T. turgidum* spp. *durum* accessions.

### Domestication may adopt riboSNitches in shaping translational subgenome asymmetry

**Fig. 6:**
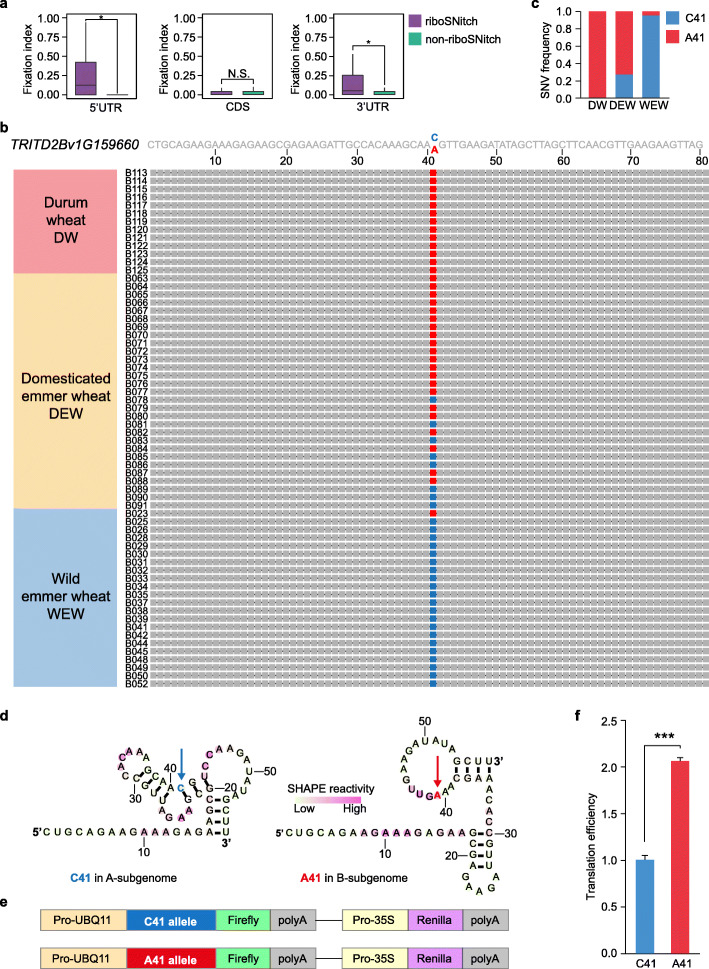
Domesticated riboSNitch modulates translation. **a** Boxplot showing the fixation index (F_ST_) of riboSNitch SNVs and non-riboSNitch SNVs at different genic regions. Significant differences between the F_ST_ of riboSNitch or non-riboSNitch groups are indicated. *P* values by Wilcoxon rank-sum test, *P < 0.05, N.S. not significant. **b** Alignment of the 5′UTR of *TRITD2Bv1G159660* in 64 tetraploid wheat accessions including durum wheat (DW, B113 to B125, 13 accessions), domesticated emmer wheat (DEW, B063 to B091, 29 accessions) and wild emmer wheat (WEW, B023 to B052,22 accessions). Sequences of different accessions were extracted from the VCF dataset of the study of Zhou et al. [13]. The sequence at position 41 with Cytosine (C41) or Adenine (A41) is colored in blue or red, respectively. **c** Bar plot showing the frequency of C41 or A41 in DW, DEW, or WEW, in the sequences shown in Fig. 6b. **d** SHAPE-directed RNA structure models of C41 allele in the A subgenome and A41 allele in the B subgenome, with C41 and A41highlighted using the arrows colored in blue and red, respectively. **e** Schematic of the plasmid design for the dual luciferase reporter assay of C41 allele and A41 allele. The 5′UTR of *TRITD2Bv1G159660* with C41 or A41 were fused upstream of the Firefly luciferase coding sequence; Renilla luciferase was used as an internal control. **f** Comparison of translation efficiency of *TRITD2Bv1G159660* C41 and A41 alleles by dual luciferase reporter assay for the design shown in Fig. 6e. *** indicates *P* < 0.01, by Student’s *t*-test, *n* = 8, error bars indicate se

Durum wheat (DW), *T. turgidum* ssp. *durum*, evolved from domesticated emmer wheat (DEW); *T. turgidum* ssp. *dicoccum*, is a major cereal grain used for pasta and couscous production. DEW was derived from wild emmer wheat (WEW), *T. turgidum* ssp. *dicoccoides*, in the Fertile Crescent about 10,000 years ago [12]. We examined whether the 3564 riboSNitches are differentiated in these three sub-species (DW, DEW, and WEW). We calculated the fixation index (F_ST_) to measure the species differentiation for these riboSNitches [43]. We found that F_ST_ values of riboSNitches were significantly higher than those of non-riboSNitches in both 5′UTR and 3′UTR, suggesting a stronger selection of riboSNitches over non-riboSNitches across these three subspecies (Fig. 6a). The F_ST_ value of riboSNitches was very similar to that of non-riboSNitches in CDS, implying the absence of strong selection (Fig. 6a). Thus, our results suggest the riboSNitches in 5′UTR and 3′UTR, but not in CDS, were more strongly selected during domestication in comparison with non-riboSNitches.

We then asked whether these riboSNitches selected through domestication impact on translational subgenome asymmetry between homoeologs. To avoid additive functions from multiple riboSNitches, we selected one differentiated riboSNitch located at position 41 in the 5′UTR of the homoeologous pair *TRITD2Av1G193730* and *TRITD2Bv1g159660*. In the A subgenome homoeolog, the riboSNitch denotes the sequence of Cytosine (C41), while in the B subgenome homoeolog, it denotes the sequence of Adenine (A41) (Fig. 6b). We examined the sequence variation in all sequenced DW accessions and found that this riboSNitch remains the same across all the DW accessions, with C41 in the A subgenome and A41 in the B subgenome (Fig. 6b, c). We further examined the sequences of both homoeologs in the DEW and WEW populations. We found a difference on the nucleotide frequency of the B subgenome A41 nucleotide, comparing the B subgenome A41 nucleotide in all DW accessions, 28% DEW accessions and 95% WEW accessions harbor C41 (Fig. 6b, c). This suggests a progressive selection from C to A at position 41 in the B subgenome during domestication.

This C41/A41 riboSNitch caused significant differences of RNA structure between A and B subgenome homoeologs in our SHAPE-Structure-seq (Fig. 6d). For the A subgenome homoeolog, the C41 formed into a C-G base pair with G22, resulting in a much stronger RNA structure (Fig. 6d). Whereas, for the B subgenome homoeolog, the A41 was unpaired, contributing to a weaker RNA structure (Fig. 6d). Notably, the TE of the A subgenome was significantly lower than that of the B subgenome (listed in Additional file 2: Table S1). Mindful of the different effect of 5′UTR mRNA structure on translational subgenome asymmetry (Fig. 3b), we then assessed the impact of this riboSNitch on translation using the dual luciferase reporting assay (Fig. 6e). We fused the 5′UTR with C41 or A41 on the upstream of Firefly coding sequence, respectively, and used an identical Renilla luciferase as an internal control for both designs (Fig. 6e). We then determined the translation efficiency for each allele and found that the TE of A41 allele was significantly higher than that of the C41 allele with over a two-fold increase (Fig. 6f, *P* < 10^-10^). Therefore, this C41/A41 riboSNitch was sufficient to significantly alter translational levels, indicating that this riboSNitch contributes to the translational subgenome asymmetry of the homoeologous pair *TRITD2Av1G193730* and *TRITD2Bv1g159660*. Taken together, our results suggest that riboSNitches may have been adopted in modulating translational subgenome asymmetry during wheat domestication.

## Discussion

In this study, we generated the translatome and RNA structurome for tetraploid wheat and discovered subgenome asymmetry in translation, similar to previously reported asymmetry in gene content and expression [14, 44–46]. Moreover, our in vivo RNA structure landscape of tetraploid wheat revealed that RNA structure played an important role in modulating translational subgenome asymmetry. Our results suggest that human selection during wheat domestication affected translation asymmetry via RNA structure variation.

### Translational subgenome asymmetry is another level for understanding wheat functional innovation

Allopolyploidization frequently induces a series of rapid genetic and epigenetic modifications as a result of conflicts between parental genomes [47, 48]. Consequently, genome structure and gene expression changes often lead to one subgenome having more retained genes (a ‘biased fractionation’ resulting in a dominant vs recessive genome) and exhibiting higher expression and genome dominance [7]. This biased fractionation feature played a vital role in the evolution of polyploid organisms. The availability of high-quality plant genomes has yielded an improved mechanistic understanding of subgenome dominance and implications for agricultural, ecological, and evolutionary research [46, 49–52]. Recent findings regarding the uneven evolution of the genome in shaping allopolyploidy mainly focus on genome and transcriptome levels [51, 53, 54]. Similar research has addressed functional subgenome bias in allopolyploid wheat. For example, detailed gene expression atlases of hexaploid wheat showed that ~30% of homoeologous triads with non-balanced expression patterns reflect the adaptive plasticity, possible the result of neo- or subfunctionalization [14].. However, only a defined group of mRNAs that succeed in translation reflect the functional genome readout. The mechanism underpinning translation regulation may offer one explanation of the differences between the transcriptome and translatome. Previous studies in other species showed that translation efficiency does not scale linearly with mRNA levels, resulting in a weak positive correlation [16, 55]. Thus, studying translation is a critical step in understanding gene expression regulation. Here, we obtained the wheat translatome using polysome profiling, proven to be a powerful tool for addressing the translational status of mRNAs [56]. We chose an important cereal, durum wheat, to establish the translatome data in an allopolyploid organism. Similar to previous studies in other species, we observed only a weak correlation of (*r*= 0.21, *P* < 10^-16^) between TE and RNA abundance in wheat (Fig. 1b). For most genes, the transcriptional level may not represent the translational level, emphasising the critical role of translational regulation in gene expression.

Among the factors suggested to affect translation, we found a strong GC content impact, as evidenced by a positive correlation of 0.57. Consistent with the observation in rice [21], our results suggested that species with GC-rich genomes, such as rice and wheat, may favor efficient translation of genes with high GC content. In assessing the impact of codon usage, we used both CAI and tAI [17, 18]. CAI defines the relative adaptiveness of an individual codon encoding a given amino acid, while tAI also considers the availability of tRNA at each codon along the gene [19]. Surprisingly, we found a positive correlation between CAI and TE, but a very poor correlation between tAI and TE (Fig. 1d, and Additional file 1: Figure S2A). In general, the advantage of using tAI over CAI is that tAI combines both codon frequency and the efficiencies of each codon-anticodon pairing [18, 19]. Based on our tAI measurement, codon preference does not seem to have a primary role in modulating translation. Future study in other polyploid plants may help to understand whether polyploidy would result in weak impact of codon preference on translation.

Previous reports at the transcriptional level show that extremely biased homoeologous expression occurred rapidly upon polyploidization in allotetraploid wheat populations [46]. At the translation level, we found that more than half of tetraploid wheat homoeologs showed significant translation asymmetry (Fig. 1e and Additional file 2: Table S1) in wheat seedlings, suggesting that expression asymmetry also explicitly occurred at the translation level. The associated biological functions of the A subgenome homoeologs with higher TEs are distinct from those of the B subgenome homoeologous genes with higher TEs; the former homoeologous group related to development, cell wall, nucleic acid metabolism, photosynthesis, and primary metabolism functions, while the latter group of genes related to rRNA processing, abiotic stress response, biotic stress response, redox regulation, secondary metabolism, and cytoskeletal organization (Fig. 1f). Homoeologous genes with different biological functions show different translation levels. This implies some factors like environmental changes may have influenced the functional differentiation of subgenomes in allopolyploids during evolution and adaptation [57, 58]. Therefore, in our study on durum wheat, selection seems to have favored some homoeologous genes from B subgenome with higher translational levels in stress response, redox regulation, and secondary metabolism context. In contrast, when some homoeologous genes from the A subgenome show a higher translational level, then vital functions, such as nucleic acid metabolism, photosynthesis, and primary metabolism predominate.

### In vivo RNA structure maintains structural flexibility in polyploid wheat

Compared to the transcriptome size in *Arabidopsis* and rice, the tetraploid wheat transcriptome is vast (fewer than 30,000 for *Arabidopsis*, 40,000–50,000 for rice, and over 66,000 of high confidence genes in tetraploid wheat) [12, 59, 60]. For our in vivo RNA structurome, we acquired over 2.8 billion reads of 150-nt pair-end reads to achieve nucleotide-resolution coverage of over 34,018 transcripts, i.e., over half of expressed genes in durum wheat (Additional file 1: Figure S4D). This dataset enabled us to comprehensively explore general RNA structure features.

By comparing in vivo RNA structures with in silico predicted RNA structures which are predicted to be the most thermostable RNA structures, we found that in vivo RNA structures of most mRNAs in durum wheat were very different from in silico predicted RNA structures (Fig. 2c), as observed in other species including *Arabidopsis*, rice, yeast, human cells, mouse, and zebrafish [24, 26, 31, 61, 62]. Cellular factors may have greatly contributed to the differences of RNA structure between in vivo and in silico. These folding differences were due to a greater extent of single-strandedness, suggesting in vivo RNA structures remain structurally flexible. Maintenance of this RNA structural flexibility may ensure the fulfilment of biological processes such as translation. Notably, the change of cellular structure was suggested to be altered during polyploidization [47]. It will be of great interest to investigate in the future how in vivo RNA structures changed during the polyploidization.

### RNA structure plays an important role in modulating translational subgenome asymmetry

In general, mRNAs with more single-strandedness along with a high magnitude of 3-nt periodicity of the structure pattern in the CDS region tend to be highly translated (Fig. 3a). Our results in durum wheat are consistent with previous observations in both *Arabidopsis* and rice [26, 31]. A moderate anti-correlation between base pairing probability and translation efficiency in both 5′UTR and CDS regions suggested that RNA structure is another important player in modulating translation, along with GC content (Fig. 3b, c). A relatively weaker correlation in CDS than that in 5′UTR may be due to the impact of ribosomes on destabilizing RNA structures in CDS [22].

Considering that GC content affects RNA secondary structure based on thermodynamics [39, 63, 64], our assessment of the relationship between GC content and RNA structure revealed that only 3′UTR mRNA structure moderately correlates with GC content (Additional file 1: Figure S5C), indicating that GC content does not significantly affect in vivo RNA structure. Similar to the observation in rice, another GC-rich monocot, our results suggest *i*n vivo RNA structures in GC-rich species tend to maintain structural flexibility [31]. The poor association between RNA structure and GC content (Additional file 1: Figure S5) implies that the impact of RNA structure on translation is likely to be independent of GC content. The GC content is likely to affect the preference of certain tRNAs, thereby, translation, rather than via in vivo RNA structure.

For transcription asymmetry, both gene-body CG methylation and histone modifications were suggested to be key factors in common wheat [14]. Several factors have been identified as involved in translational regulation such as GC content and codon preference; however, we did not observe significant contributions from both GC content and codon preference for translation asymmetry (Additional file 1: Figure. S2B, C), most likely due to the high degree of sequence similarity between the A and B subgenomes. These observations led us to explore another intrinsic property of RNA, RNA structure. Our discovery of a positive correlation between RNA structural difference and TE difference (Fig. 4a) explained why RNA structure is an important factor in modulating translational subgenome asymmetry, among other factors such as GC content and codon preference.

mRNAs involved in biological functions related to abiotic and biotic stress responses, responses to metal ion and phytohormone signaling, preferred this RNA structure-mediated translational subgenome asymmetry (Fig. 4d). This preference may be one of the driving forces for wheat functional innovation. Notably, factors such as temperature, salt, reactive oxygen species induced by plant pathogens, metal ion, and phytohormone signaling, involved in these biological functions were suggested to generally affect RNA structure folding [65–67]. Thus, it is likely that mRNAs with these biological functions tend to adopt RNA structures in response to different elements to modulate their translational levels and shape subgenome asymmetry. Therefore, this RNA structure-mediated translational subgenome asymmetry may be dynamic in response to varying environmental changes such as temperature and metal ion concentration, which will be an exciting perspective to follow in future research.

### RiboSNitches functioning as an RNA structural switch is globally conserved within durum wheat cultivars

RiboSNitches were first proposed to interpret human disease-associated SNVs located on individual UTRs or non-coding RNAs [40]. Recently, a genome-wide RNA structure study showed that riboSNitches occur at a genome-wide scale, around 2000 riboSNitches were found between mother and father in a human family [27]. Our study determined 3564 riboSNitches out of 304,835 SNVs between pairs of homoeologs in durum wheat (Fig. 5b and Additional file 1: Figure S8C, D). Overall, roughly 1.17% of SNVs led to significant RNA structural changes. However, there may be more SNVs with potential as riboSNitches since RNA structure alters dynamically under different conditions or developmental stages. We found that slightly more riboSNitches on A-G and/or G-A transition, mostly due to higher frequency of transition SNVs (Fig. 5b, Additional file 1: Figure S8C, D). We also found that riboSNitches were preferably located in 5′UTR (Fig. 5c), suggesting their potential role in regulating translation. Additionally, it is worth emphasizing that wheat homoeologs share the same cellular environment. RNA structural differences are solely affected by SNVs, rather than other varying cellular conditions, such as metabolites and cations.

The fundamental elements of RNA structure are the nucleotide pairs involved in making base pairs (G-C and A-U) [68]. To maintain a helix, selection pressure on these base-pairing nucleotides was imposed during evolution [69]. Sequence conservation at a base-pairing site tends to be much higher than that at a single-stranded site, such as a loop or bulge [70]. Significant RNA structural changes in riboSNitches were alterations from base-pairing to single-strandedness. Thus, we investigated the conservation of these riboSNitches across durum wheat populations and found that riboSNitches were more conserved than non-riboSNitches (Fig. 5d). Our results suggested that these SNVs, acting as RNA structural switches, are highly evolved, to ensure precise functional consequences of RNA structure-mediated regulation, such as translation.

### Domestication-shaped riboSNitches offer a new avenue for molecular breeding research

Through domestication humans have influenced the evolution of the wheat genome via selected breeding of favored agronomic traits [12]. For example, domesticated emmer wheat (DEW) is derived from wild emmer wheat (WEW) and was further evolved by human selection to generate a variety of cultivars around the world, such as durum wheat (DW). Compared to WEW and DEW, many different genetic signatures exist in DW, of which SNVs are significant and easily detected, and thus, frequently used in breeding selection and association studies [12, 13]. However, most selected SNVs were in non-coding regions such as UTRs [71], which limited the number of functional SNVs for altering the codon. In this study, we reveal the presence of a high number of riboSNitches in wheat (1.17% of all SNVs examined), and more importantly, we discover a stronger selection of riboSNitches compared to non-riboSNitches across WEW, DEW, and DW subspecies (Fig. 6a). This suggests that riboSNitches might have had an important role in modulating the translational landscape of wheat during domestication. Moreover, riboSNitches offer a new perspective for SNVs application studies such as genome-wide association studies (GWAS), offering new data interpretation opportunities, particularly for those selected SNVs located at non-coding regions and/or synonymous sites. Our results indicate that utilization of riboSNitches during evolution may be parallel to or earlier than amino acid evolution, supporting the RNA world theory [72, 73].

Finally, we validated functional associations with these selected riboSNitches. We determined that a single riboSNitch between two homoeologs in the 5′UTR was sufficient to switch RNA structures, subsequently leading to significant differences at the translational level in a reporter assay (Fig. 6d–f). This riboSNitch was highly conserved within durum wheat cultivars but divergent in domesticated emmer wheat and wild emmer wheat accessions (Fig. 6b, c). Our results suggest that this riboSNitch may have been selected during domestication to modulate translational subgenome asymmetry between homoeologs. Therefore, our work offers the baseline for pursuing whether riboSNitches have potential as a new molecular breeding tool in wheat and polyploid crop improvement strategies.

## Conclusions

Translational subgenome asymmetry occurs in tetraploid wheat, with ~55% of homoeologs showing non-balanced translation efficiencies. RNA structure plays a prevalent role in contributing to this translational subgenome asymmetry. We identified 3564 riboSNitches between the two subgenomes that had arisen upon polyploidization or during domestication, which might contribute to shaping the translation landscape of wheat. Our first translatome and in vivo RNA structurome of polyploid wheat presented here provides the context and basis for future studies of polyploid evolution and for developing new strategies for wheat molecular breeding.

## Methods

### Plant and growth conditions

The tetraploid wheats (2*n* = 4*x* = 28, BBAA) used in this study were *Triticum turgidum* ssp. *durum* cv. Kronos (durum wheat). Wheat seeds of tetraploid durum wheat cv “Kronos” were obtained from the John Innes Centre Germplasm Resource Unit. The seeds were washed with 70% ethanol for 30 s for surface sterilization, followed by washing 3 times with distilled water. The seeds were then soaked in distilled water for 24 h and plated to Petri dishes with 3 layers of wet filter paper. The Petri dishes were placed in a growth chamber at 22°C in the dark for 4 days, with distilled water added every day to keep the filter paper wet.

### Plasmid construction

Single-stranded DNA (ssDNA) fragments of 5′UTRs of *TRITD2Bv1g159660* with the A41 or C41 allele were synthesized by Integrated DNA Technologies. Double-stranded DNA (dsDNA) fragments were amplified using CloneAmp HiFi PCR Premix (Clontech). PCR products were further introduced into the expression vector inter2 digested with *BamH*I and *Sma*I using In-Fusion (Clontech). The sequences of ssDNA and primers are listed in Additional file 6: Table S5.

### Polysome extraction and separation

Polysome profiling was performed as described with optimization [74]. Briefly, approximately 500mg wheat seedlings were harvested and ground into fine powder in liquid nitrogen. 500 μl precooled polysome extraction buffer (200 mM Tris-HCl, pH 8.4, 50 mM KCl, 1% deoxycholic acid, 25 mM MgCl2, 2% Polyoxyethylene 10 tridecyl ether, 2 mM DTT, 400 U/mL recombinant Rnasin, 50 μg/mL cycloheximide) was added and incubated on ice for 30 min for full lysis. The lysate was centrifuged at 13,200 rpm for 15 min at 4°C, and 500 μl supernatant was loaded on top of a 15–60% sucrose gradient. Then, the gradient tubes were balanced and started the ultracentrifugation run at 50,000 rpm for 3 h in a Beckman MLS-50 rotor at 4°C. After centrifugation, the gradients were applied to a Piston Gradient Fractionator (BioComp, Canada), continuously reading the eluate at 254 nm with a Fraction Collector (Gilson, USA) paired with FlowCell software (BioComp, Canada). Gradients that proceeded by mechanical navigation of fractionation were collected in a total of 16 fractions. Polysomes were measured by calculating the area under the curve between the disome to the end of the polysomal region (outlined in Fig. 1a) and dividing by the total absorbance profiles. Finally, fractions representing the translation level were subjected to RNA isolation with TRIzol reagent (Ambion). To generate a total RNA control, seedlings were directly applied to RNA extraction using Qiagen RNeasy plant mini kit. RNAs extracted were used for library generation by BGI Genomics following the manufacture’s BGISEQ-500 protocol.

### Chemical probing of RNA structure in tetraploid Kronos

The SHAPE chemical 2-methylnicotinic acid imidazolide (NAI) was synthesized as described [34]. In vivo SHAPE probing was performed following our previous study in *Arabidopsis* with modifications [75]. Briefly, the etiolated seedlings were harvested by carefully removing the remaining seed and incubated with 200 mM NAI at 22°C for 15 min. NAI was quenched using 5 times DTT amount to that of NAI and washed 3 times with distilled water. To generate a control without NAI probing (−NAI), the same volume of DMSO as that for NAI was added to the incubation buffer. The washed seedlings were then harvested and ground into fine powder in liquid nitrogen and subjected to RNA extraction using RNeasy Plant Mini Kit (Qiagen).

### Library generation for SHAPE-Structure-seq

Libraries of SHAPE-structure-seq were prepared as described [28]. PolyA-selected RNA was recovered and reverse-transcribed using SuperScript III First-Strand Synthesis System (Thermofisher) and RT primer (5′CAGACGTGTGCTCTTCCGATCTNNNNNN3′). cDNA ligation was performed using Circligase ssDNA Ligase (Epicentre) to ligate the 3′ end of cDNA to a ssDNA linker (5′-PhosNNNAGATCGGAAGAGCGTCGTGTAG-/3SpC3/3′) for 12 h at 65°C. Ligation product over 100 nt was recovered using QIAquick Gel Extraction Kit (Qiagen) after separation using UREA-TBE 10% Gel (Invitrogen). Purified ligated cDNA was amplified using KAPA Library Amplification Kits (Roche) with Forward Library primer (5′AATGATACGGCGACCACCGAGATCTACACTCTTTCCCTACACGACGCTCTTCCGATCT3′) and Reverse Library primers (5′CAAGCAGAAGACGGCATACGAGATNNNNNNGTGACTGGAGTTCAGACGTGTGCTCTTCCGATC3′, where NNNNNN denotes the barcodes, e.g., Index1 is CGTGAT for Illumina sequencing). The PCR products were purified using agarose gel to recover 200~650 bp fragments using QIAquick Gel Extraction Kit (Qiagen). Sequencing was performed to generate 150 nt pair-end reads using the Illumina Hiseq4000 platform.

### Gel-based analysis of SHAPE probing

One microgram of the total RNA of −NAI or +NAI probing was dissolved in 6 μl water; 1 μl of Cy5 labeled RT primer (5 μM) for 18S rRNA (listed in Additional file 6: Table S5) and 0.5 μl of 10 mM dNTPs were added and further denatured at 95°C for 3 min. After cooling down to 50°C, 2 μl of 5X RT buffer (100 mM Tris (pH 8.3), 500 mM LiCl, 15 mM MgCl_2_, 5 mM DTT), and 0.5 μl Superscript III (Thermofisher) was added. Incubation at 50°C for 20 min was allowed for cDNA synthesis, followed by incubation at 85°C for 10 min to inactivate Superscript III. 0.5 μl of NaOH (2M) was added and incubated at 95°C for 10 min to degrade the cDNA hybridized RNA. 10 μl of 2X stopping dye (95% formaldehyde, 20 mM EDTA (pH8.0), 20 mM Tris (pH 7.5), orange G) was added and incubated at 95°C for 5 min, and further kept on 65°C until loading to 8% Acrylamide:Bis-Acrylamide-Urea gel for electrophoresis. For the sequencing lanes, unprobed RNA was dissolved in 5 μl water and 1 μl 10mM of corresponding ddNTP (Roche) was added at the beginning of the reaction. The gel was scanned using Typhoon FLA 9500 system (GE Healthcare) and quantified using ImageQuant; SHAPE reactivity was normalized using a 2–8% standard method [76].

### Dual luciferase reporting assay

Sequencing-confirmed vectors were transformed to *Agrobacterium tumefaciens* GV3101 and infiltrated into leaves of 3–4-week-old tobacco *N. benthamiana*. After 48 h of agroinfiltration, 10 mg of leaf discs were harvested and ground into fine powder in liquid nitrogen, following homogenization in Passive Lysis Buffer (PLB, Promega). The lysate was centrifuged at 13,000 rpm for 1 min before diluting the clear supernatant 20 times with PLB. The diluted supernatant was subjected to dual luciferase assay using the Dual-Luciferase Reporter Assay System (Promega) according to the manufacturer’s manual. To quantify the mRNA abundance of Firefly luciferase (F-luc) or Renilla luciferase (R-luc), RNA isolated with TRIzol or RNeasy Plant Mini Kit was digested using RNase-free TURBO™ DNase (Ambion). cDNA was synthesized using SuperScript III First-Strand Synthesis System (Thermofisher). Quantitative PCR was performed using a LightCycler 480 SYBR Green I Master (Roche) using CFX96 Touch Real-Time PCR Detection System (BIORAD) according to the manufacturer’s protocol. Translation efficiency (TE) of dual luciferase assay was calculated as described [77, 78]. Protein level or mRNA abundance of F-luc was normalized to that of internal control R-luc. The normalized F-luc protein level was scaled relative to normalized mRNA abundance to generate the raw translation efficiency for both the C allele and the A allele. Relative translation efficiency was further calculated through normalization to that of the raw translation efficiency of the C allele. Primers used are listed in Additional file 6: Table S5.

### Library mapping and data processing

The raw reads were filtered through both quality control and adaptor trimming. The trimmed reads were mapped to durum wheat genome assembly (Svevo RefSeq 1.0) [12] using HISAT2 version 2.1.0 [79]. Then, the Kronos composite genome was made by calling SNV variants between Svevo and Kronos using freebayes-1.3.1 [80] and Genome Analysis Toolkit [81]. Due to the lack of UTR regions for a large number of genes, due to the limited annotation, we improved the durum annotation for the UTR regions using our RNA-seq reads. Homoeologous gene pairs were extracted from EnsemblCompara database [82]. All libraries were mapped to the transcriptome. Translation efficiencies for individual gene models were calculated as previously described [33]. SHAPE reactivity was calculated by subtracting from the reverse transcription stops of (+) SHAPE library to those in (−) SHAPE library, as previously described [30].

### Comparison of SHAPE-Structure-seq data to the phylogenetic structure model of 18S rRNA

Given the absence of rRNA phylogenetic structure models for the tetraploid durum wheat cultivar, Kronos, we used the 18S rRNA structure model in *Arabidopsis thaliana* which shares high sequence similarity [83]. We calculated the true positive rate which is the number of nucleotides with high in vivo SHAPE reactivities corresponding to single-stranded regions in the phylogenetic structure. We also calculated the true negative rate, calculated as the number of nucleotides with low in vivo SHAPE reactivity in our dataset corresponding to double-stranded regions in the phylogenetic structure [30].

### Calculations of tAI and CAI

The tRNA adaptation index (tAI) was calculated using the formula:

$$ tAI={\left(\prod \limits_{k=1}^n{w}_k\right)}^{\raisebox{1ex}{$1$}\!\left/ \!\raisebox{-1ex}{$n$}\right.} $$

where *w*_*k*_ denotes the relative adaptiveness of codon *k* [18] and *n* denotes the length of CDS.

The codon adaptation index (CAI) was calculated using the formula:

$$ {r}_i=\frac{f_i}{\max \left({f}_j\right)}\kern0.5em i,j\in \left[\mathrm{synonymous}\ \mathrm{codons}\ \mathrm{for}\ \mathrm{amino}\ \mathrm{acid}\right] $$

$$ CAI={\left(\prod \limits_{i=1}^n{r}_i\right)}^{\raisebox{1ex}{$1$}\!\left/ \!\raisebox{-1ex}{$n$}\right.} $$

where *f*_*i*_ denotes the frequency of codon *i* and max(*f*_*j*_) denotes the most frequent synonymous codon amino acid *j* [17].

### Identification of SNVs and riboSNitches between homoeologous gene pairs

To identify single-nucleotide variations (SNV) between homoeologous gene pairs, local sequence alignment was performed using the “Mafft” tool [84]. Bases annotated with “N” or without annotation were removed. Experimental Structure Disruption Coefficients (eSDC) were calculated to evaluate the structure disruption of SNVs, as previously described [27]. We determined riboSNitches with high confidence by measuring the significant differences of both BPP and SHAPE reactivity [27, 42]. For measuring the selection of riboSNitches, we obtained the complete genomic variation information of wheat accessions, including 13 accessions durum wheat, 29 accessions of domesticated emmer wheat and 28 accessions of wild emmer wheat [13].The “VCFtools” was endorsed to extract data from genomic variation data of different populations (including Durum and wild emmer) [85]. We then calculated the major allele frequency of each SNV in different sub-populations and generated the F_ST_ value of each SNV [43]. The F_ST_ value was calculated according to the following equation:

$$ {F}_{ST}=\frac{{\sigma_{\pi}}^2}{\pi \left(1-\pi \right)} $$

where *π* is the mean allele frequency and *σ*_*π*_^2^is the variance in allele frequency among populations [43].

To calculate the conservation ratio, we firstly calculated the frequency of each nucleotide of a given SNP from 13 tetraploid *T. turgidum* spp. Durum groups using the VCF files [13, 85].

$$ {f}_i=\frac{c(i)}{\sum_{i=1}^Nc(i)} $$

where *N* represents the number of different nucleotides observed at the polymorphism and *c* denotes the counts. The nucleotide with the maximum allele frequency on a given SNP is regarded as the major allele.

$$ {f}_{\mathrm{major}}=\max \left({f}_i\right) $$

For individual transcript, conservation ratio of riboSNitch SNP denotes to the rate of riboSNitch SNP counts which are major alleles, regarding to the total counts of riboSNitch SNPs. The conservation ratio of non-riboSNitch SNP denotes to the rate of non-riboSNitch SNP counts which are major alleles, regarding to the total counts of non-riboSNitch SNPs.

$$ \mathrm{Conservation}\ \mathrm{ratio}=\frac{c\left(\mathrm{major}\right)}{c\left(\mathrm{total}\right)} $$

### Statistical hypothesis testing

All analyses were performed using R programming language (http://www.r-project.org). Statistical methods and significance are indicated in the main text or figure legends for individual tests.

## Supplementary information

**Additional file 1: Figure S1.** The high reproducibility of the libraries for RNA-seq and polysome-seq in tetraploid Kronos. **Figure S2.** Relationship between translation efficiency and translation related factors. **Figure S3.** NAI probing of tetraploid Kronos RNA structure in vivo. **Figure S4.** The high reproducibility of the SHAPE-Structure-seq libraries. **Figure S5.** Relationship between in vivo RNA structure and GC content in different genic regions. **Figure S6.** RNA structures of homoeologous genes with differences of translation efficiency (TE) in A and B subgenome. **Figure S7.** Comparison of SHAPE reactivities in vivo for homoeologous pairs in wheat. **Figure S8.** SNV affects RNA structure *in vivo*.

**Additional file 2: TableS1.** Translation efficiency of homoeologs of A and B subgenome in tetraploid Kronos.

**Additional file 3: TableS2.** Base-pairing probability of RNA structures of homoeologs of A and B subgenome in tetraploid Kronos.

**Additional file 4: TableS3.** The list of single nucleotide variations between homoeologs of A and B subgenome in tetraploid Kronos.

**Additional file 5: TableS4.** The list of riboSNitch between homoeologs of A and B subgenome in tetraploid Kronos.

**Additional file 6: TableS5.** Primers used in this study.

**Additional file 7.** Review history.

## Data Availability

Sequence data have been deposited in the Sequence Read Archive (SRA) (https://www.ncbi.nlm.nih.gov/sra) under BioProject ID number PRJNA723219 [86].
