# Supplementary material for: Wheat in vivo RNA structure landscape reveals a prevalent role of RNA structure in modulating translational subgenome expression asymmetry
Source: Genome Biol. 2021 Nov 30;22:326. doi: 10.1186/s13059-021-02549-y (PMC8638558; doi:10.1186/s13059-021-02549-y)
Supplement: Supplementary file 1 — Additional file 1: Figure S1. The high reproducibility of the libraries for RNA-seq and polysome-seq in tetraploid Kronos. Figure S2. Relationship between translation efficiency and translation related factors. Figure S3. NAI probing of tetraploid Kronos RNA structure in vivo. Figure S4. The high reproducibility of the SHAPE-Structure-seq libraries. Figure S5. Relationship between in vivo RNA structure and GC content in different genic regions. Figure S6. RNA structures of homoeologous genes with differences of translation efficiency (TE) in A and B subgenome. Figure S7. Comparison of SHAPE reactivities in vivo for homoeologous pairs in wheat. Figure S8. SNV affects RNA structure in vivo. [file 13059_2021_2549_MOESM1_ESM.docx]

**Supplementary figures for**

**Yang et al., Wheat in vivo RNA structure landscape reveals a prevalent role of RNA structure in modulating translational subgenome expression asymmetry, *Genome Biology* (2021).**

**FigureS1**

**
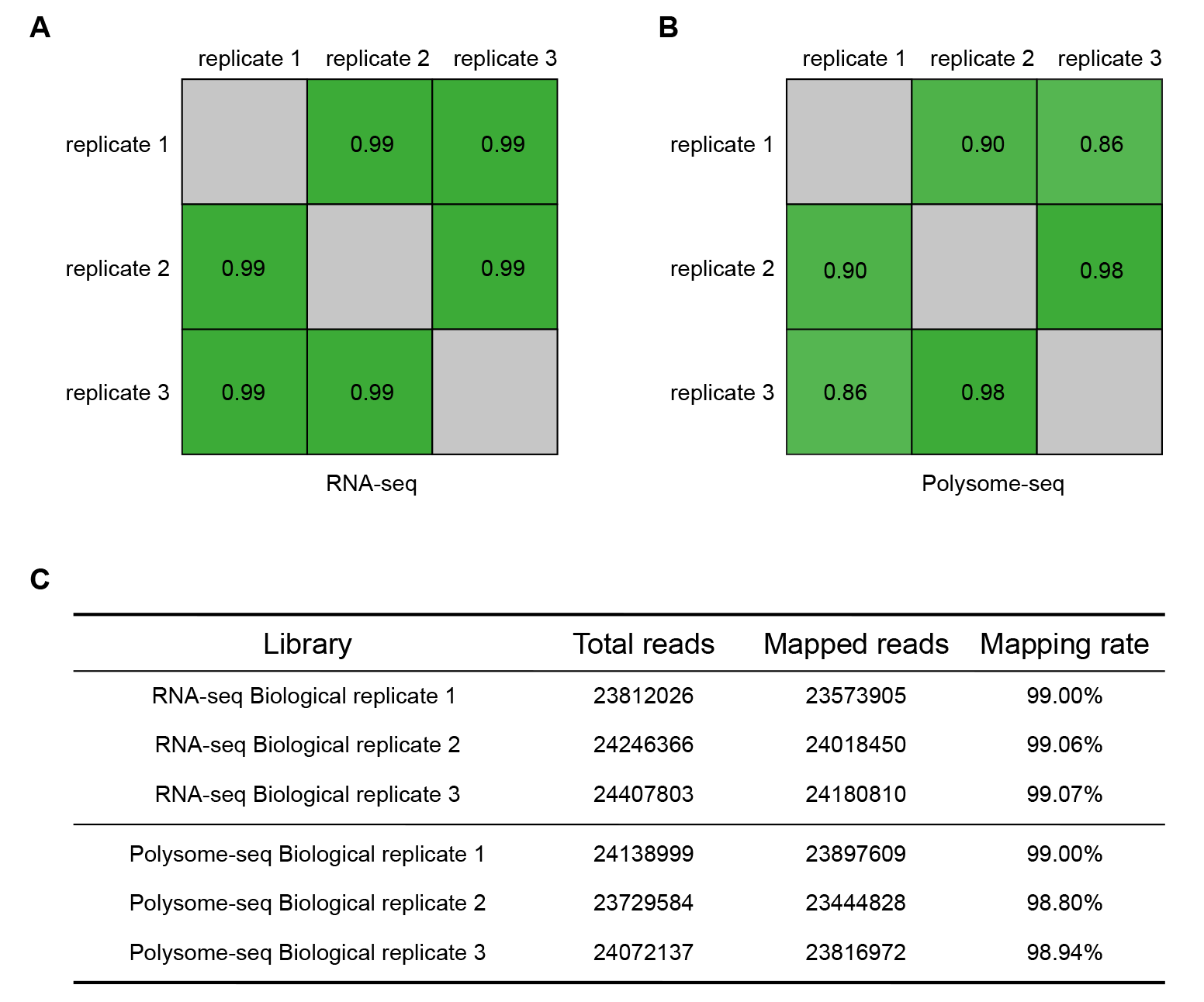
**

**Figure S1** The high reproducibility of the libraries for RNA-seq and polysome-seq in tetraploid Kronos.

**(A)** Heat plot showing the Pearson correlation coefficient among three biological replicates of RNA-seq libraries.

**(B)** Heat plot showing the Pearson correlation coefficient among three biological replicates of polysome-seq libraries.

**(C)** Statistics of the RNA-seq and polysome-seq libraries.

**FigureS2**


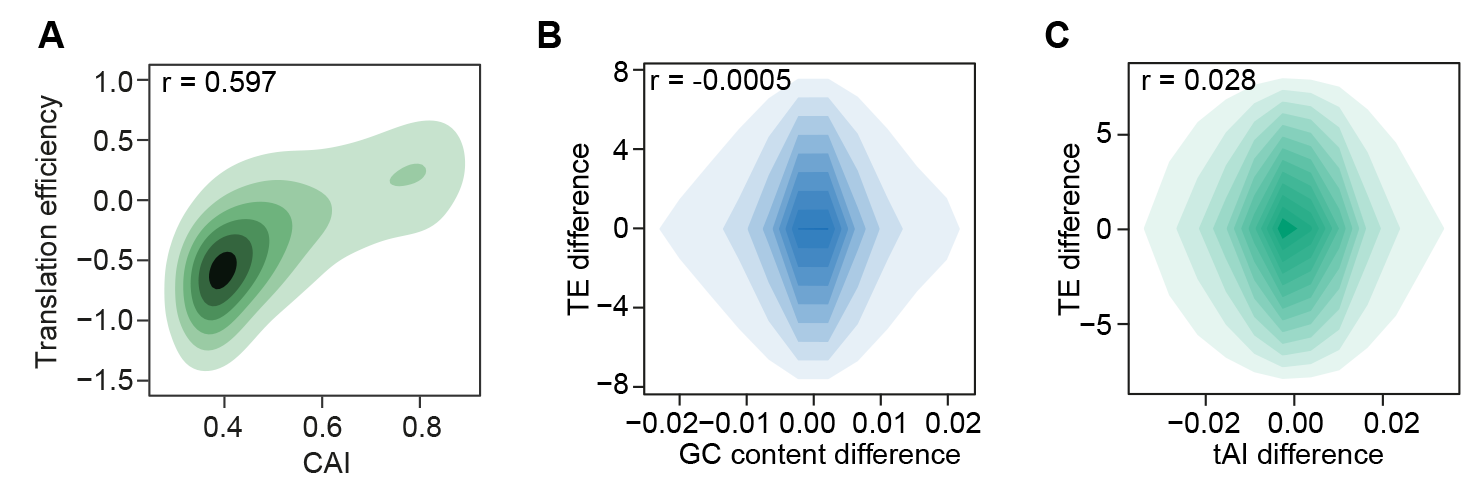


**Figure S2** Relationship between translation efficiency and translation related factors.

**(A)** Correlation between translation efficiency and CAI, with a correlation coefficient of 0.597.

**(B-C)** Scatter plot showing the correlation between the differences of translation efficiency and GC content **(B)** and tAI **(C)** in A subgenome and B subgenome homoeologous genes.

**Figure S3**


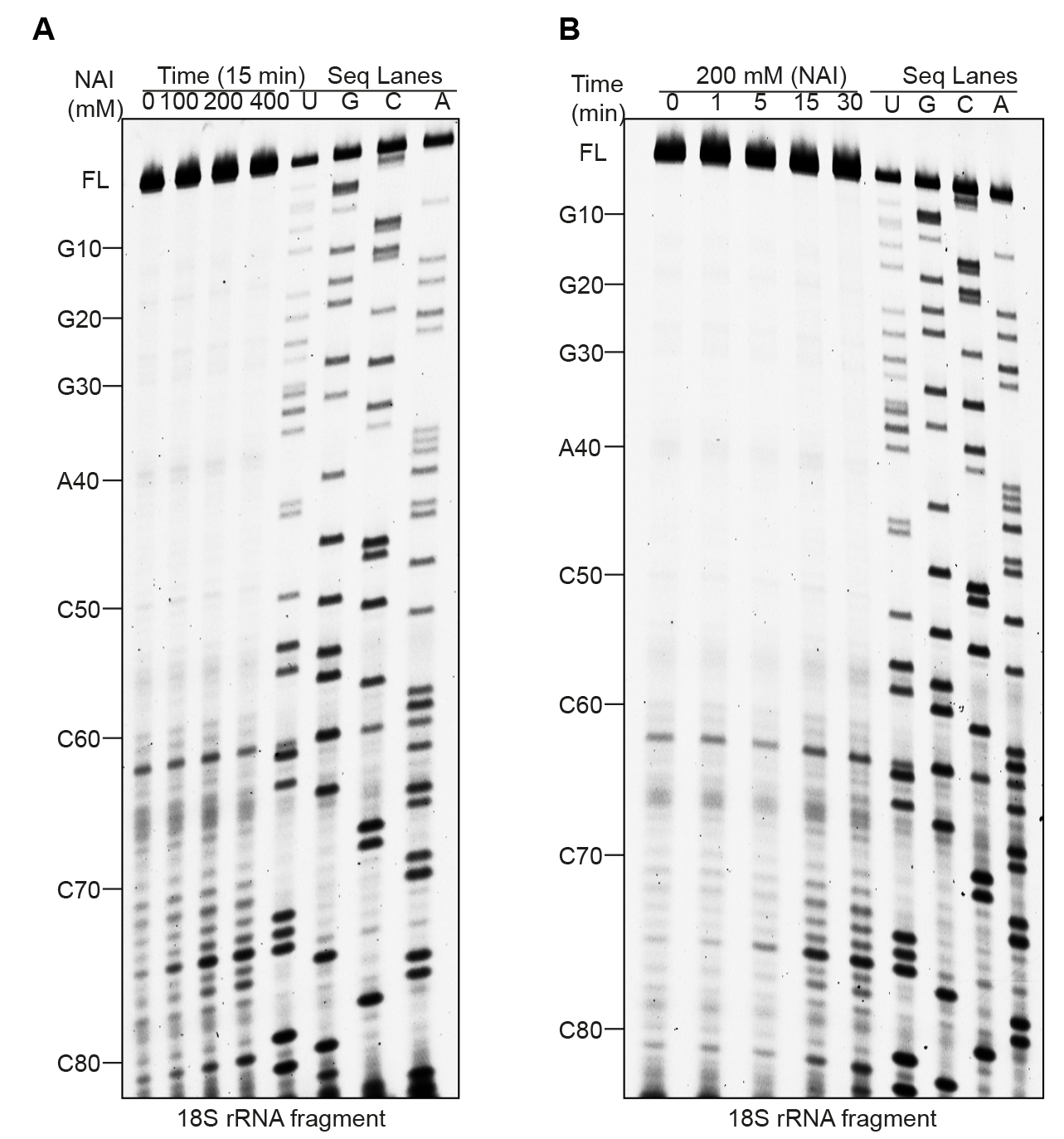


**Figure S3** NAI probing of tetraploid Kronos 18S rRNA structure *in vivo*.

**(A)** NAI modification on 18S rRNA with different concentrations of NAI over 15 minutes. The modification signal was captured using reverse transcription stalling and gel-based analysis. The Seq Lanes of A/C/G/U indicate the sequence of the corresponding stalling.

**(B)** 200 mM NAI modification on 18S rRNA with different incubation times, otherwise in A.

**FigureS4**


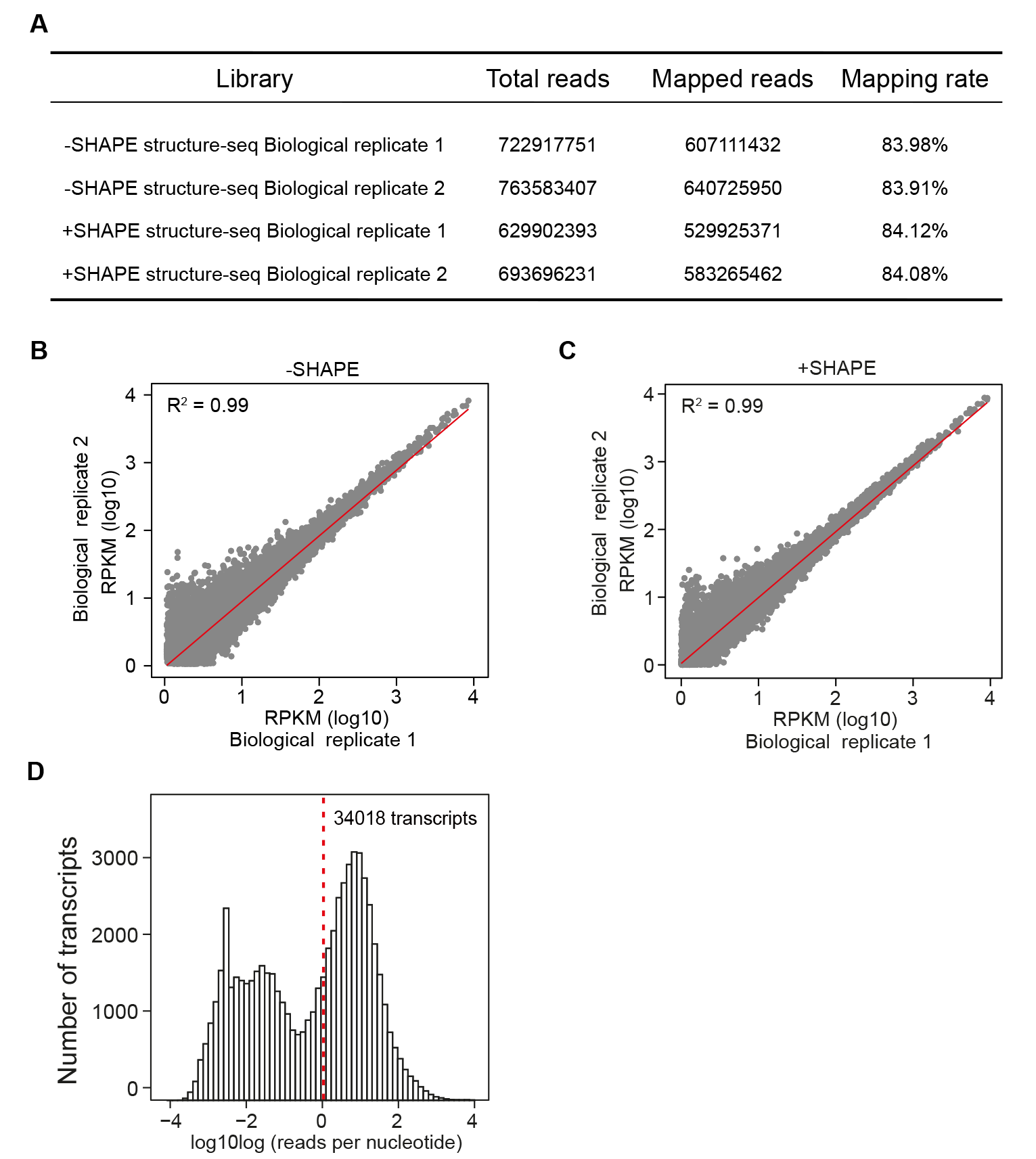
**Figure S4** The high reproducibility of the SHAPE-Structure-seq libraries.

**(A)** Statistics of our SHAPE-Structure-seq libraries.

**(B)** Scatter plot showing the Pearson correlation coefficient (PCC) between two biological replicates of -SHAPE libraries.

**(C)** Scatter plot showing the Pearson correlation coefficient (PCC) between two biological replicates of +SHAPE libraries.

**(D)** Histogram plot showing the high coverage of SHAPE-Structure-seq libraries in the Kronos transcriptome, with a sequencing depth of >1reads/nt on 34,018 transcripts, represented by our +SHAPE library.

**FigureS5**


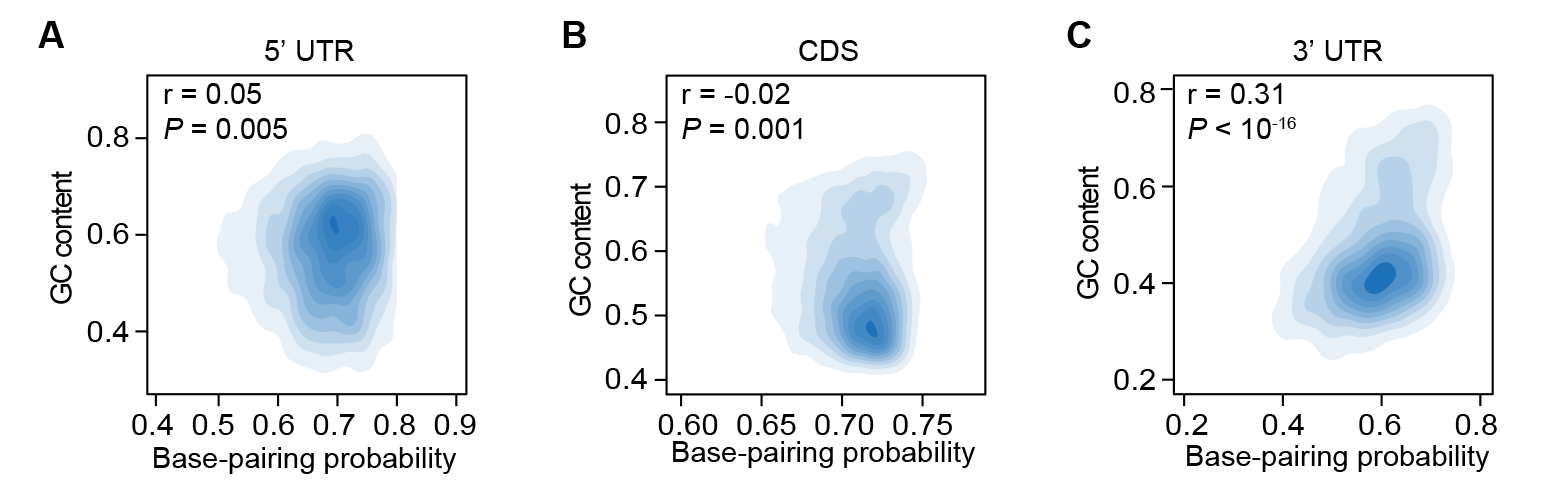


**Figure S5** Relationship between *in vivo* RNA structure and GC content in different genic regions.

**(A-C)** The scatter plots showing the correlation between base-pairing probability and GC content in 5’UTR (A), CDS (B) and 3’UTR (C), respectively.

**FigureS6**

**
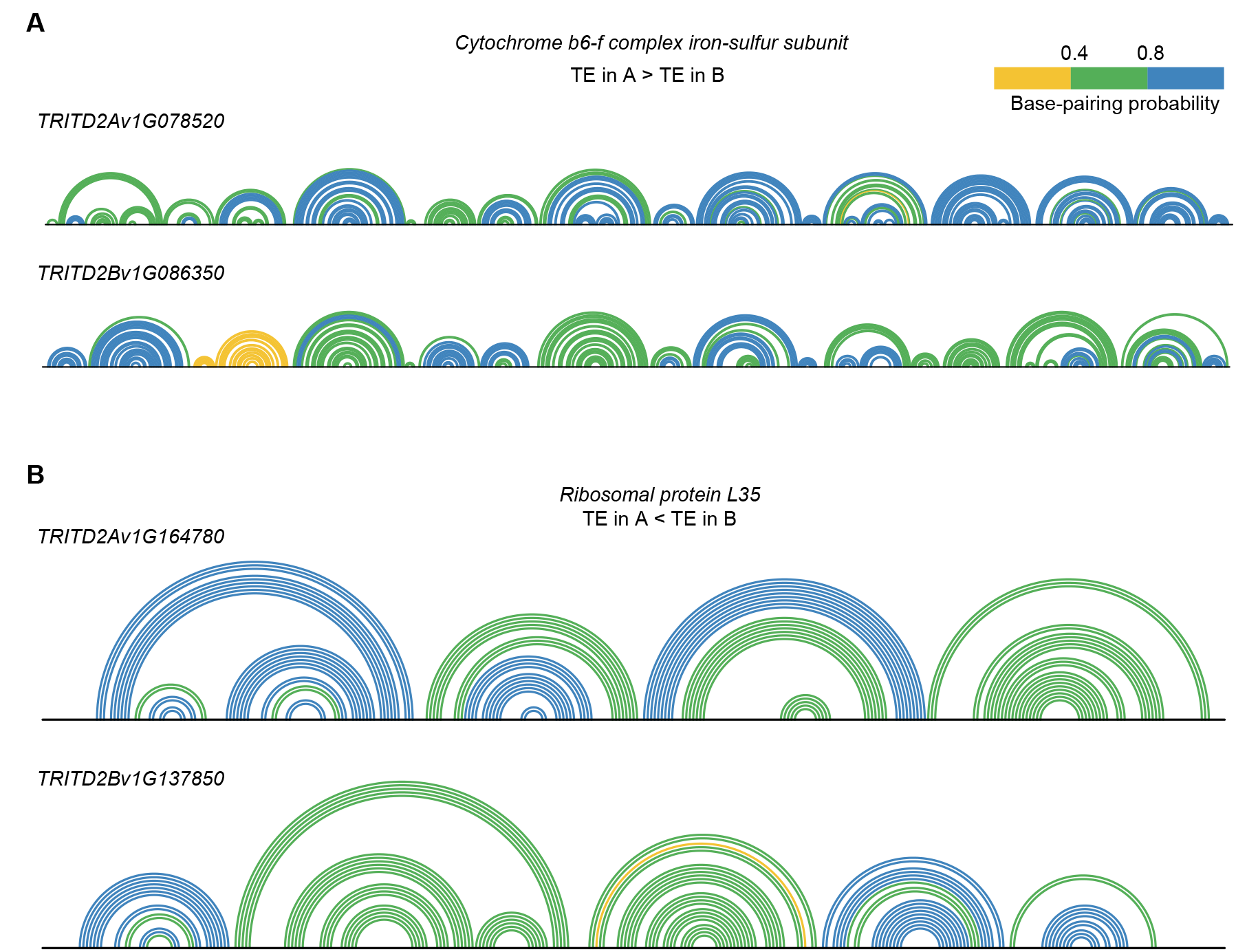
**

**Figure S6** RNA structures of homoelogous genes with differences of translation efficiency (TE) in A and B subgenome.

**(A)** Arc diagram showing the RNA structure of the homoeologous gene pair of *cytochrome b6-f complex iron-sulfur subunit*, *TRITD2Av1G078520* in A subgenome and *TRITD2Bv1G086350* in B subgeome, with higher TE in A subgenome than that in B subgenome. Every arc corresponds to one base pair, the pairing nucleotides with pairing probability lower than 0.4, between 0.4 and 0.8, or over 0.8.are indicated by lines coloured with yellow, green or blue, respectively.

**(B)** Arc diagram showing the RNA structure of the homoeologous gene pair of *ribosomal protein L35*, *TRITD2Av1G164780* in A subgenome and *TRITD2Bv1G137850* in B subgeome, with lower TE in A subgenome than that in B subgenome. Otherwise in Figure S6A.

**Figure S7**


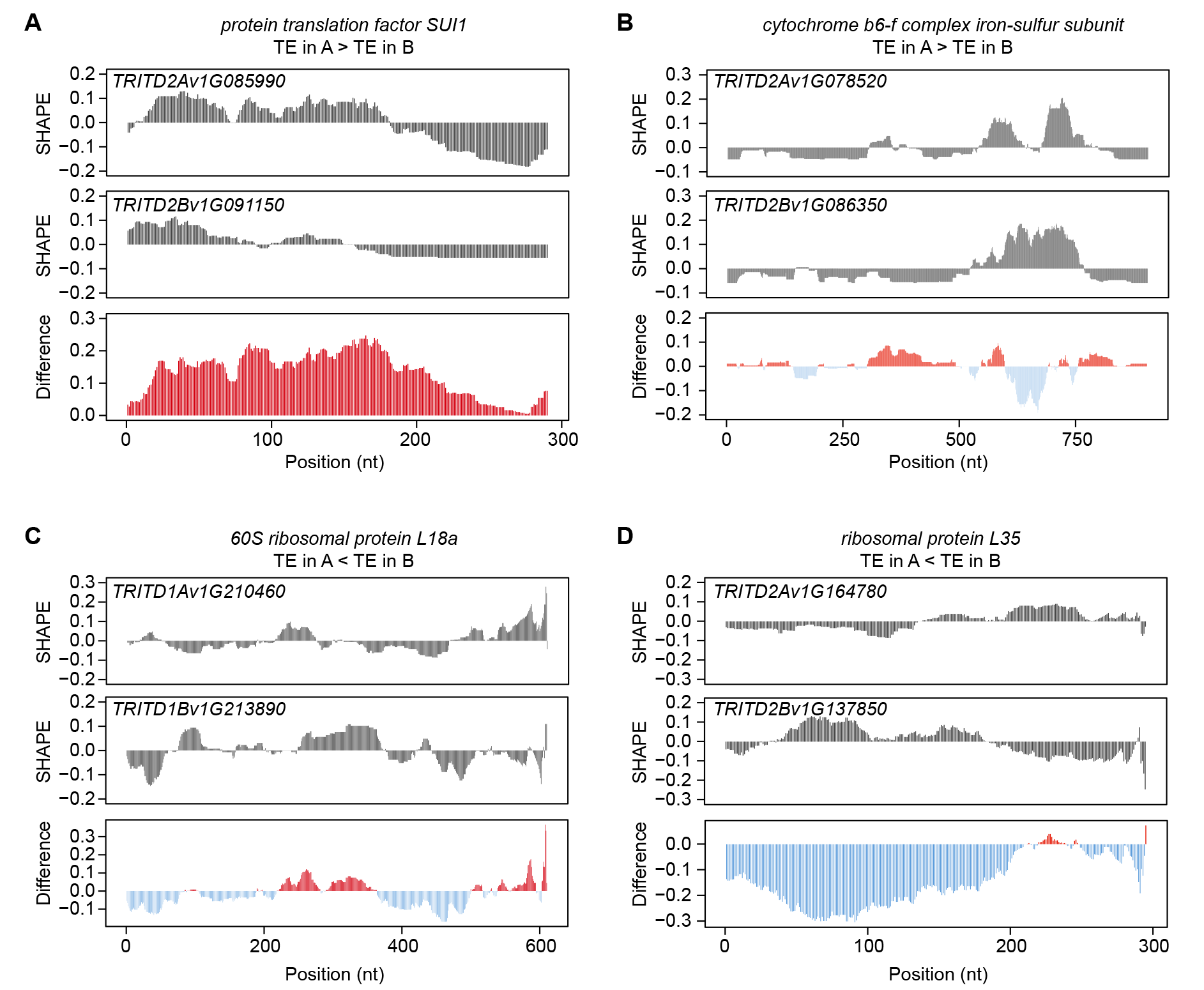


**Figure S7 Comparison of SHAPE reactivities *in vivo* for homoeologous pairs in wheat.**

**(A)** Normalized SHAPE reactivity profile *in vivo* for the homoeologous pair: *protein translation factor SUI1*, *TRITD2Av1G085990* in the A subgenome and *TRITD2Bv1G091150* in the B subgenome, with higher TE in the A subgenome than in the B subgenome.

**(B)** Normalized SHAPE reactivity profile *in vivo* for the homoeologous pair: *cytochrome b6-f complex iron-sulfur subunit*, *TRITD2Av1G078520* in the A subgenome and *TRITD2Bv1G086350* in the B subgenome, with higher TE in the A subgenome than in the B subgenome.

**(C)** Normalized SHAPE reactivity profile *in vivo* for the homoeologous pair: *60S ribosomal* *protein L18a*, *TRITD1Av1G210460* in the A subgenome and *TRITD1Bv1G213890* in the B subgenome, with lower TE in the A subgenome than in the B subgenome.

**(D)** Normalized SHAPE reactivity profile *in vivo* for the homoeologous pair: *ribosomal protein L35*, *TRITD2Av1G164780* in the A subgenome and *TRITD2Bv1G137850* in the B subgenome, with lower TE in the A subgenome than in the B subgenome.

**FigureS8**

**
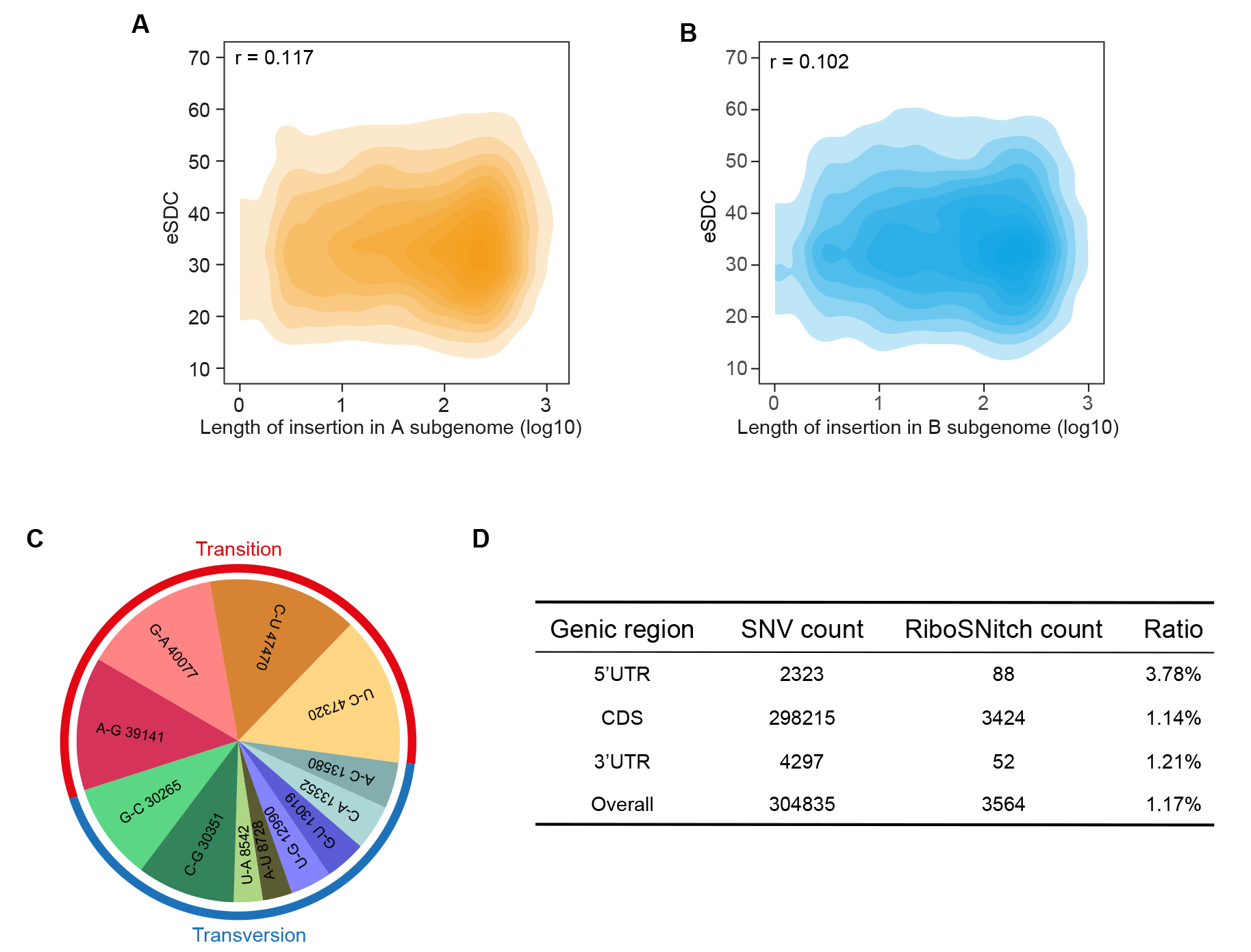
**

**Figure S8 SNV affects RNA structure *in vivo*.**

**(A)** Scatter plot showing the correlation between the length of insertion in A subgenome and experimental structural disruption coefficient (eSDC).

**(B)** Scatter plot showing the correlation between length of insertion in B subgenome and experimental structural disruption coefficient (eSDC).

**(C)** Pie plot showing the counts of nucleotide transition or transversion for single nucleotide variation (SNV) between the A and B subgenomes in tetraploid Kronos.

**(D)** Statistics of SNV count, riboSNitch count and the ratio of riboSNitch relative to SNV, in different genic regions.
