# Supplementary material for: Wheat in vivo RNA structure landscape reveals a prevalent role of RNA structure in modulating translational subgenome expression asymmetry
Source: Genome Biol. 2021 Nov 30;22:326. doi: 10.1186/s13059-021-02549-y (PMC8638558; doi:10.1186/s13059-021-02549-y)
Supplement: Supplementary file 7 — Additional file 7. Review history. [file 13059_2021_2549_MOESM7_ESM.docx]

Review History

**First round of review**

**Reviewer 1**

**Are you able to assess all statistics in the manuscript, including the appropriateness of statistical tests used?** Yes, and I have assessed the statistics in my report.

**Comments to author:**

In this paper, Yang and colleagues explore the role of RNA structure in modulating subgenome asymmetry at the translational level. The quality of the work and of the presented experiments is extremely high. The findings are presented in a clear and convincing way.

I would really like to see this manuscript published, and I have only a major concern, that, however, I would like to see the authors addressing in their revised manuscript, and this concerns the way structural analyses have been performed. All the structural analyses presented by the authors are based on the "interpretation" of the SHAPE signal, by using this as a constraint to drive structure prediction (i.e. the calculation of BPPs, the in vivo vs. in silico comparison, etc.). This approach introduces large biases, as it heavily relies on the ability of thermodynamics-driven structure prediction tools to correctly interpret the experimental constraints. For example, the choice of the slope and intercept parameters is crucial when performing experimentally-informed structure modelling. What slope and intercept values did the authors choose and why? To avoid these issues, I would like the authors to repeat their analyses, in particular the ones comparing homoelogs, by directly comparing the SHAPE reactivities. A way to account for the different lengths of different homoelogs, would be to use sequence alignment between 2 homoelogs, to align the SHAPE profiles to each other.

Furthermore, rather than comparing the in vivo-derived structure to the in silico structure, it would be much more interesting to understand whether the same asymmetry reported for the homoelogs in vivo, is also present when refolding the RNA in vitro, or whether this is something that is regulated by in vivo-specific factors. This is not essential, as I can understand the cost of such an experiment might be prohibitive, but it would provide additional insights into the phenomenon here described. Maybe this can be done for 2-3 selected RNAs?

**Reviewer 2**

**Are you able to assess all statistics in the manuscript, including the appropriateness of statistical tests used?** No, I do not feel adequately qualified to assess the statistics.

**Comments to author:**

In their work entitled "Wheat in vivo RNA structure landscape reveals a prevalent role of RNA structure in modulating translational subgenome expression asymmetry", Yang and colleagues have explored the relationships between translation efficiency and mRNA structure in wheat. By comparing the translation efficiency (measured by estimating ribosome association), and the mRNA structure at the whole genome level, they conclude that mRNA structure has a strong impact on translation efficiency (TE) in wheat.

This work represents an important resource for the community by providing a translatome and structurome of wheat mRNAs, and gives very interesting insight on the determinants of translation efficiency. Overall the data are quite compelling, and goes very nicely from genome-wide approach to the functional validation of the impact of mRNA structure on one selected candidate gene.

I have however a few comments that I think should be addressed to make the article easier to follow, and to further support the authors' conclusions. One first very general comment is that the methods for statistical analyses or calculation of some indexes are not sufficiently described. For example how was the conservation ratio used on Figure 5 calculated? How was the F(st) calculated? (authors do refer to a paper, but it describes different methods to estimate it). Likewise for the dual luciferase assay, how was TE calculated? Likewise CAI and tAI should be better described, as readers will not necessarily be familiar with these parameters.

1- In the abstract, and in the introduction L.49, authors write that mRNA structure "modulates" TE. Maybe the world modulate is a bit strong as it would imply that the mRNA structure can change and that this would modify TE in return, "modulating" implies the possibility of tuning the process, which is not explored here (and wouldn't be easy to explore). Maybe better to phrase it as mRNA structure has a strong impact on TE. Another instance where the text should maybe be toned down is in the discussion. Authors write that a "moderate" anti-correlation between base pairing probability and TE suggested that RNA structure is a "crucial" player in modulating translation. If the correlation is moderate, than maybe the phenomenon is not that crucial (which does not mean it is irrelevant).

2- In Figure 1, and actually throughout the manuscript, authors study individual correlation relationships between TE and various parameters (GC content, codon usage, mRNA structure etc..). Would there be a way of analyzing these data with a PCA or similar to determine what part of the variability is actually explained by each parameter?

3- Figure 2: what is the overlap between the 3 gene sets seen on panel f (BPP higher in subgenome A, higher in subgenome B or not different between sub-genomes) and the gene sets defined on Fig 1 e (TE higher in subgenome A, higher in subgenome B or not different). Does it significantly differ from what one by chance?

3- L214-215 I am not sure I quite get why authors are interested in the 3-nt periodicity of SHAPE reactivity. Can they explain a bit more the point they are trying to make here?

4- L215 and after, BPP actually refers to the average BPP for each gene, maybe best to specify this?

5- Authors mention in several places in the manuscript that GC content positively correlates with TE? but never discuss what could be the explanation for this observation.

6- L 257-263: On Figure 4, it is true that the mRNA with the highest TE seems to have the weakest structure, but only if one looks at the 5' region. If one compares the 3', it's actually the opposite. Maybe better to specify this in the text.

7- L 267-270. The notion the homeologs of genes involved in specific cellular processes might favour RNA-structure mediated translational regulation is interesting but highly speculative. Notably, the use of "regulation" would imply that the structure can be regulated, and there is no evidence for this in the paper.

8- L 314-315, authors write the "ribosnitches were highly enriched in the 5'UTR region compared to those in the CDS and 3'UTR regions". However this is not supported by any kind of statistical analysis (what would be the expected values? Is the enrichment really significant?)

9- Figure 5d. How vas the conservative ratio measured? Isnt it surprising that seemingly nearly identical values observed in the CDS differ with a very low p-value (indicated by ***), whereas much more obvious differences in the 3'UTR appear to be less significant?

10- In the discussion, authors highlight a number of interesting observations, but do not really offer an explanation for them. For example, how come CAI but not tAI correlates well with TE? Genes with different TE between the A and B subgenomes belong to distinct GO categories, are those the same that would be found by comparing mRNA abundance? How come the in vivo mRNA structure is so poorly predicted in silico? How does GC content affect TE?

**Authors Response**

**Point-by-point response to Reviewers**

**Reviewer #1:**

I*n this paper, Yang and colleagues explore the role of RNA structure in modulating subgenome asymmetry at the translational level. The quality of the work and of the presented experiments is extremely high. The findings are presented in a clear and convincing way.*

**RESPONSE**

We thank the Referee for their appreciation of our paper:*“The quality of the work and of the presented experiments is extremely high. The findings are presented in a clear and convincing way.”* We are very appreciative of the time and effort that the Referee has spent assessing our work and for their suggested improvements. We have fully addressed all the comments and improved our manuscript accordingly.

**Comment 1**

*I would really like to see this manuscript published, and I have only a major concern, that, however, I would like to see the authors addressing in their revised manuscript, and this concerns the way structural analyses have been performed. All the structural analyses presented by the authors are based on the "interpretation" of the SHAPE signal, by using this as a constraint to drive structure prediction (i.e. the calculation of BPPs, the in vivo vs. in silico comparison, etc.). This approach introduces large biases, as it heavily relies on the ability of thermodynamics-driven structure prediction tools to correctly interpret the experimental constraints. For example, the choice of the slope and intercept parameters is crucial when performing experimentally-informed structure modelling. What slope and intercept values did the authors choose and why? To avoid these issues, I would like the authors to repeat their analyses, in particular the ones comparing homoelogs, by
directly comparing the SHAPE reactivities. A way to account for the different lengths of different homoelogs, would be to use sequence alignment between 2 homoelogs, to align the SHAPE profiles to each other.*

**RESPONSE**

We are very grateful to the Referee for their constructive suggestions. The reason we calculated BPPs is because BPP presents the likelihood of single-strandedness, whereas SHAPE reactivity indicates the modification efficiency. The folding parameters we used are the default settings in the *RNAfold* module (slope m: 1.9; intercept b: -0.7), showing high performance in RNA structure modeling consistent with other studies[1, 2].

We followed the Referee’s suggestion and repeated our analyses based on SHAPE reactivities, with our results shown in Rebuttal Figure 1. As expected, our SHAPE reactivity results were in high agreement with those based on BPP. We found a significant correlation between average SHAPE reactivity and TE in both 5’UTR and CDS regions, with correlation coefficients of 0.27 and 0.41, respectively (Rebuttal Figures 1A-B), indicating mRNAs with weaker structures in the 5’UTR or CDS regions tend to be highly translated (Rebuttal Figures 1A-B). Average SHAPE reactivity in 3’UTR shows lower correlation with the translation efficiencies (TEs), suggesting 3’UTR structures have less effect on translation (Rebuttal Figure 1C). We also found that SHAPE reactivity differences positively correlated with TE differences, whereby the bigger the difference of RNA structure between homoeologs, the greater the difference of TEs (Rebuttal Figure 1D), agreeing with the result shown in Fig. 4a.

**Rebuttal Figure 1 Relationship between RNA structure and translation efficiency. A-C** Scatter plots showing the correlation between translation efficiency and SHAPE reactivity across 5’UTR, CDS and 3’UTR, respectively. **D** Scatter plot showing the correlation between translation efficiency difference of homoeologs and SHAPE reactivity of homoeologs of the full-length transcript.

**Rebuttal Figure 2 Comparison of SHAPE reactivities *in vivo* for homoeologous pairs in wheat. A** Normalized SHAPE reactivity profile *in vivo* for the homoeologous pair: *protein translation factor SUI1*, *TRITD2Av1G085990* in the A subgenome and *TRITD2Bv1G091150* in the B subgenome, with higher TE in the A subgenome than in the B subgenome. **B** Normalized SHAPE reactivity profile *in vivo* for the homoeologous pair: *cytochrome b6-f complex iron-sulfur subunit*, *TRITD2Av1G078520* in the A subgenome and *TRITD2Bv1G086350* in the B subgenome, with higher TE in the A subgenome than in the B subgenome. **C** Normalized SHAPE reactivity profile *in vivo* for the homoeologous pair: *60S ribosomal* *protein L18a*, *TRITD1Av1G210460* in the A subgenome and *TRITD1Bv1G213890* in the B subgenome, with lower TE in the A subgenome than in the B subgenome. **D** Normalized SHAPE reactivity profile *in vivo* for the homoeologous pair: *ribosomal protein L35*, *TRITD2Av1G164780* in the A subgenome and *TRITD2Bv1G137850* in the B subgenome, with lower TE in the A subgenome than in the B subgenome.

As suggested, we plotted the corresponding SHAPE reactivity profiles for the illustrated homoelogs (Rebuttal Figure 2), that show high agreement with the RNA structure models in our manuscript, elucidating the relationship between RNA structure difference and TE difference. *Protein translation factor SUI1* in the A subgenome with a TE significantly higher than that in the B subgenome also had greater single-strandedness in its A subgenome RNA structure and higher SHAPE reactivities compared to that in the B subgenome (Rebuttal Figure 2A). In contrast, *60S ribosomal protein L18a* in the A subgenome with TE values significantly lower than those in the B subgenome had less single-strandedness in its A subgenome RNA structure with lower SHAPE reactivities, compared to that in the B subgenome (Rebuttal Figure 2C). SHAPE profiles of additional homoeologous pairs are shown in Rebuttal Figures 2B and 2D, corresponding to the RNA structure models in Figures S6A-B. The profiles of SHAPE reactivities of homoeologs in Rebuttal Figure 2 are shown in New Figure S7 in the revised manuscript.

**Comment 2**

*Furthermore, rather than comparing the in vivo-derived structure to the in silico structure, it would be much more interesting to understand whether the same asymmetry reported for the homoelogs in vivo, is also present when refolding the RNA in vitro, or whether this is something that is regulated by in vivo-specific factors. This is not essential, as I can understand the cost of such an experiment might be prohibitive, but it would provide additional insights into the phenomenon here described. Maybe this can be done for 2-3 selected RNAs?*

**RESPONSE**

We thank the Referee for this comment. Our previous studies, alongside others in multiple species, have shown that *in vitro* RNA structure is more like the *in silico* predicted thermostable RNA structure, which is very different from the *in vivo* RNA structure [3, 4]. Before generating the *in vivo* RNA structurome, we probed individual wheat 18S rRNA (Rebuttal Figures 3A-B) for its *in vitro* and *in vivo* RNA structures. Consistent to previous reports in other species[5, 6], the *in vivo* SHAPE profile is very different from the corresponding *in vitro* SHAPE profile, with a correlation coefficient of 0.41(Rebuttal Figure 3C). Thus, our work focused on the *in vivo* RNA structurome, allowing a broad investigation of the functional roles of RNA structure in living plants.

For the Referee’s interest, we sequenced our *in vitro* libraries. Due to the large size of the wheat transcriptome, the sequencing depth of the *in vitro* libraries is relatively lower than the *in vivo* libraries, resulting in 3204 transcripts with nucleotide-resolution RNA structure information. We found that the *in vivo* RNA structural differences between the two homoeologs are very distinct when compared to the *in vitro* RNA structural differences (Rebuttal Figure 3D). The individual examples are illustrated in Rebuttal Figures 3E-H. These observations further suggest that RNA structural differences between the homoeologs in living cells are very specific, associated with their molecular functions such as translation.

**Rebuttal Figure 3 Comparison of SHAPE reactivities *in vitro* for homoeologous pairs in wheat. A-B** NAI probing of tetraploid Kronos 18S rRNA structure *in vivo* and *in vitro*. NAI modification was performed at 22 °C with 150 mM NAI for 15 min *in vivo*, or 150 mM NAI for 5 min *in vitro*. The modification signal was captured using reverse transcription stalling and gel-based analysis. The Seq Lanes of A/C/G/U indicate the sequence of the corresponding stalling. **C** Scatter plot showing the relationship between *in vivo* signal and *in vitro* signal, with the signals shown in Rebuttal Figures 3A-B, the raw signal of individual nucleotide was normalized using the total signal of all nucleotides of the whole lane, resulting in the relative signal. **D** Scatter plot showing relationship between differences of *in vivo* SHAPE reactivities of homoeologs and differences of *in vitro* SHAPE reactivities of homoeologs. **E** Normalized SHAPE reactivity profiles *in vitro* for the homoeologous pair: *protein translation factor SUI1*, *TRITD2Av1G085990* in the A subgenome and *TRITD2Bv1G091150* in the B subgenome, with higher TE in the A subgenome than in the B subgenome. **F** Normalized SHAPE reactivity profiles *in vitro* for the homoeologous pair: *cytochrome b6-f complex iron-sulfur subunit*, *TRITD2Av1G078520* in the A subgenome and *TRITD2Bv1G086350* in the B subgenome, with higher TE in the A subgenome than in the B subgenome. **G** Normalized SHAPE reactivity profiles *in vitro* for the homoeologous pair: *60S ribosomal* *protein L18a*, *TRITD1Av1G210460* in the A subgenome and *TRITD1Bv1G213890* in the B subgenome, with lower TE in the A subgenome than in the B subgenome. **H** Normalized SHAPE reactivity profiles *in vitro* for the homoeologous pair: *ribosomal protein L35*, *TRITD2Av1G164780* in the A subgenome and *TRITD2Bv1G137850* in the B subgenome, with lower TE in the A subgenome than in the B subgenome.

**Reviewer #2:**

*In their work entitled "Wheat in vivo RNA structure landscape reveals a prevalent role of RNA structure in modulating translational subgenome expression asymmetry", Yang and colleagues have explored the relationships between translation efficiency and mRNA structure in wheat. By comparing the translation efficiency (measured by estimating ribosome association), and the mRNA structure at the whole genome level, they conclude that mRNA structure has a strong impact on translation efficiency (TE) in wheat.*

*This work represents an important resource for the community by providing a translatome and structurome of wheat mRNAs, and gives very interesting insight on the determinants of translation efficiency. Overall the data are quite compelling, and goes very nicely from genome-wide approach to the functional validation of the impact of mRNA structure on one selected candidate gene.*

**RESPONSE**

We are very thankful to the Referee for their appreciation of our work as articulated above. We are grateful for the time and effort that the Referee has spent in helping us strengthen our manuscript. We have fully addressed all the comments as follows:

**Comment 1**

*I have however a few comments that I think should be addressed to make the article easier to follow, and to further support the authors' conclusions.*

*One first very general comment is that the methods for statistical analyses or calculation of some indexes are not sufficiently described. For example how was the conservation ratio used on Figure 5 calculated? How was the F(st) calculated? (authors do refer to a paper, but it describes different methods to estimate it). Likewise for the dual luciferase assay, how was TE calculated? Likewise CAI and tAI should be better described, as readers will not necessarily be familiar with these parameters.*

**RESPONSE**

We thank the Referee for these comments. We have added the details below into the method section of our revised manuscript, from lines 701-706, lines 730-739, lines 753-771.

To calculate the conservation ratio, we first calculated the frequency of each nucleotide of a given SNP from 13 tetraploid T.turgidum spp. Durum groups using the VCF files [7, 8].

$$f_{i}=\frac{c(i)}{\sum_{i=1}^{N} c(i)}$$

Where *N* represents the number of different nucleotides observed at the polymorphism and *c* denotes the counts. The nucleotide with the maximum allele frequency on a given SNP is regarded as the major allele.

$$f_{major}=max(f_{i})$$

For individual transcript, the conservation ratio of riboSNitch SNP denotes to the rate of riboSNitch SNP counts which are major alleles, regarding to the total counts of riboSNitch SNPs. The conservation ratio of non-riboSNitch SNP denotes to the rate of non-riboSNitch SNP counts which are major alleles, regarding to the total counts of non-riboSNitch SNPs.

$$conservation ratio=\frac{c(major)}{c\left( total \right)}$$

The F_ST_ value was calculated according to the following equation:

$$F_{ST}=\frac{{\sigma_{\pi}}^{2}}{\pi(1-\pi)}$$

where $\pi$ is the mean allele frequency, ${\sigma_{\pi}}^{2}$is the variance in allele frequency among populations[9].

The translation efficiency (TE) of our dual luciferase assay was calculated as described [10, 11]. The protein level or mRNA abundance of Firefly luciferase (F-luc) was normalized to that of the internal control Renilla luciferase (R-luc). The normalized F-luc protein level was scaled relative to normalized mRNA abundance to generate the raw translation efficiency for the C allele and the A allele. The relative translation efficiency was further calculated through normalization to that of the raw translation efficiency of the C allele.

The tRNA adaptation index (tAI) was calculated using the formula:

$$tAI=\left( \prod_{k=1}^{n} w_{k} \right)^{\frac{1}{n}}$$

where *w_k_* denotes the relative adaptiveness of codon *k* [12], and *n* denotes the length of CDS.

The codon adaptation index (CAI) was calculated using the formula:

$$r_{i}=\frac{f_{i}}{\max\left( f_{j} \right)} i,j\in\left[ synonymous codons for amino acid \right]$$

$$CAI=\left( \prod_{i=1}^{n} r_{i} \right)^{\frac{1}{n}}$$

where $f_{i}$ denotes the frequency of codon *i* and $\max\left( f_{j} \right)$ denotes the most frequent synonymous codon amino acid *j* [13].

**Comment 2**

*1- In the abstract, and in the introduction L.49, authors write that mRNA structure "modulates" TE. Maybe the world modulate is a bit strong as it would imply that the mRNA structure can change and that this would modify TE in return, "modulating" implies the possibility of tuning the process, which is not explored here (and wouldn't be easy to explore). Maybe better to phrase it as mRNA structure has a strong impact on TE. Another instance where the text should maybe be toned down is in the discussion. Authors write that a "moderate" anti-correlation between base pairing probability and TE suggested that RNA structure is a "crucial" player in modulating translation. If the correlation is moderate, than maybe the phenomenon is not that crucial (which does not mean it is irrelevant).*

**RESPONSE**

We thank the Referee for their constructive suggestions. We have toned down all these words as suggested in our revised manuscript, for example, we have changed “*modulates*” to “*may have a strong impact on*” as shown in abstract page and introduction section lines 49-50, and changed “*crucial*” to “*important*” in line 502, highlighted in yellow.

**Comment 3**

*2- In Figure 1, and actually throughout the manuscript, authors study individual correlation relationships between TE and various parameters (GC content, codon usage, mRNA structure etc..). Would there be a way of analyzing these data with a PCA or similar to determine what part of the variability is actually explained by each parameter?*

**RESPONSE**

We thank the Referee for their comments. We performed the principal component analysis of TE and other variables. We found that tAI contributes the least to the principal component and BPP has the greatest effect among all other variables (Rebuttal Figure 4). The arrows of both GC content and CAI were pointed in the same direction and were close together, indicating that they have similar effects. These results are consistent with our correlation analyses: 1) BPP negatively correlated with TE; 2) The factors of GC, CAI, and RNA abundance each positively correlated with TE; 3) tAI had the least correlation with TE.

**Rebuttal Figure 4 Principal component analysis of the variables relevant to translation efficiency.** The contribution of each variable is coloured using the colour gradient as shown.

**Comment 4**

*3- Figure 2: what is the overlap between the 3 gene sets seen on panel f (BPP higher in subgenome A, higher in subgenome B or not different between sub-genomes) and the gene sets defined on Fig 1 e (TE higher in subgenome A, higher in subgenome B or not different). Does it significantly differ from what one by chance?*

**RESPONSE**

We thank the Referee for this comment. Of the13294 homoeologous pairs included for TE comparison and 7271 homoeologous pairs for BPP comparison, an overlap of 6941pairs were found, as shown in Rebuttal Figure 5A. We then classified these pairs into 9 categories according to the TE or BPP comparison between the A and B homoeologs (A<B, A=B and A>B, respectively). As listed in Rebuttal Figure 5B we saw a similar number of overlapped pairs in categories A>B and A<B, for both TE and BPP comparisons, ruling out the possibility of a significant difference by chance.

**Rebuttal Figure 5 Overview of the number of homoeologs for TE and BPP comparisons. A** Venn plot showing the overlap between homoeologous pairs included for TE and BPP comparisons, as listed in Tables S1 and S2, respectively. **B** Table showing the number of overlapped homoeologs between different categories of TE and BPP comparisons between A and B subgenomes.

**Comment 5**

*3- L214-215 I am not sure I quite get why authors are interested in the 3-nt periodicity of SHAPE reactivity. Can they explain a bit more the point they are trying to make here?*

**RESPONSE**

We thank the Referee for this comment. The 3-nt periodicity of RNA structure pattern was first predicted by *in silico* analysis of human and mouse mRNA folding, that suggested this 3-nt periodicity in mRNA secondary structure may facilitate the formation of intramolecular helices and a more compact transcript folding. This, in turn, may enhance resistance of the genetic message to degradation and modification [14]. Following this *in silico* work, a previous *in vitro* RNA structure analysis, based on structure-specific enzymes, confirmed this 3-nt periodicity RNA structure pattern [15]. Our previous study in plants, found that this 3-nt periodicity exists *in vivo* where the 3-nt periodicity RNA structure pattern only appears in the coding region and is absent in UTR regions [3]. Notably, this periodic signal was intensified in high TE transcripts and absent from low TE transcripts, suggesting that a hidden code in the *in vivo* RNA structure may influence polyribosome association and, by inference, translation [3]. To make this point easier to follow, we have added the sentence: “*We then examined the 3-nt periodicity, an RNA structure feature indicative of high translation*”, as shown in lines 214-215.

**Comment 6**

*4- L215 and after, BPP actually refers to the average BPP for each gene, maybe best to specify this?*

**RESPONSE**

We thank the Referee for this comment and have specified average BPP where needed throughout the revised manuscript with 11 changes, such as that line 177 as highlighted in yellow.

**Comment 7**

*5- Authors mention in several places in the manuscript that GC content positively correlates with TE? but never discuss what could be the explanation for this observation.*

**RESPONSE**

We thank the Referee for this comment. “*GC content positively correlates with TE*” was first found in rice, another monocot[16], where it was suggested that GC content might affect RNA structure and the selection by tRNAs. In our previous work in rice, we ruled out the effect of GC on the *in vivo* RNA structure [17]. Therefore, GC content is likely to affect the preference of certain tRNAs which will be interesting to investigate in the future. We have added more discussion on this observation, in lines 510-511, lines 513-515 highlighted in yellow in our revised manuscript.

**Comment 8**

*6- L 257-263: On Figure 4, it is true that the mRNA with the highest TE seems to have the weakest structure, but only if one looks at the 5' region. If one compares the 3', it's actually the opposite. Maybe better to specify this in the text.*

**RESPONSE**

We thank the Referee for this suggestion. We have further specified the differences of features between 5’UTR and 3’UTR, by adding the sentence: “*Of note, the link between high TE and weak RNA structure is more obvious in 5’UTR and less obvious in 3’UTR, emphasizing a predominant role of RNA structure in 5’UTR affecting translation efficiency*” in lines 267-269, highlighted in yellow in our revised manuscript.

**Comment 9**

*7- L 267-270. The notion the homoeologs of genes involved in specific cellular processes might favour RNA-structure mediated translational regulation is interesting but highly speculative. Notably, the use of "regulation" would imply that the structure can be regulated, and there is no evidence for this in the paper.*

**RESPONSE**

We thank the Referee for this comment. We have toned down the wording as suggested, with: “*We found that the homoeologs with biological functions related to abiotic stress response, biotic stress response, metal ion response and phytohormone signalling display significantly higher correlations between RNA structure difference and TE difference (Fig. 4d), implying a close link between RNA structure and translation efficiency in these homoeologs*” in our revised manuscript, as highlighted in yellow in lines 276-277.

**Comment 10**

*8- L 314-315, authors write the "ribosnitches were highly enriched in the 5'UTR region compared to those in the CDS and 3'UTR regions". However this is not supported by any kind of statistical analysis (what would be the expected values? Is the enrichment really significant?)*

**RESPONSE**

We thank the Referee for this comment. We calculated the ratio of the riboSNitches in different genic regions, and specified the results thus: “*Among different genic regions, we found that the ratio of riboSNitches in 5' UTR was over three times higher than those in CDS and 3' UTR regions*” in our revised manuscript, in lines 320-321, highlighted in yellow in our revised manuscript.

**Comment 11**

*9- Figure 5d. How vas the conservation ratio measured? Isnt it surprising that seemingly nearly identical values observed in the CDS differ with a very low p-value (indicated by ***), whereas much more obvious differences in the 3'UTR appear to be less significant?*

**RESPONSE**

We thank the Referee for this comment. For individual transcript, conservation ratio of riboSNitch SNP denotes to the rate of riboSNitch SNP counts which are major alleles, regarding to the total counts of riboSNitch SNPs. The conservation ratio of non-riboSNitch SNP denotes to the rate of non-riboSNitch SNP counts which are major alleles, regarding to the total counts of non-riboSNitch SNPs, as explained in Response to Comment 1.

The lower *P*-value in the CDS region compared to that in the 3'UTR region is mainly due to the number of identified riboSNitches (3424 riboSNitches in the CDS region and 52 riboSNitches in the 3’UTR region, as listed in Table S4).

**Comment 12**

*10- In the discussion, authors highlight a number of interesting observations, but do not really offer an explanation for them. For example, how come CAI but not tAI correlates well with TE? Genes with different TE between the A and B subgenomes belong to distinct GO categories, are those the same that would be found by comparing mRNA abundance? How come the in vivo mRNA structure is so poorly predicted in silico? How does GC content affect TE?*

**RESPONSE**

We thank the Referee for these comments. The different correlations for CAI and tAI might be due to the limitation of the current wheat genome annotation, which requires further effort from the wheat community.

Genes with different mRNA abundances between the A and B subgenomes are only partially overlapped with genes with different TEs between the A and B subgenomes (Rebuttal Figure 6A). We further performed gene ontology analysis based on the genes with different mRNA abundances between the A and B subgenomes and found a significant different enrichment of GO terms for homoeologous pairs with A > B or A < B for mRNA abundance comparison (Rebuttal Figures 6B-C). As shown, these GO categories for genes with different mRNA abundances are also quite different from those for genes with different TEs as shown in Fig. 1f.

*In vivo* mRNA structure tends to be more single-stranded than *in silico* predicted RNA structure, which is the most thermostable structure. Our observation is consistent with previous findings in yeast, mouse, human cell, rice, *Arabidopsis* and zebrafish [3, 5, 17-19]. It is likely that factors in living cells such as RNA binding proteins might affect *in vivo* RNA structure folding, leading to the differences between *in vivo* RNA structure and *in silico* predicted RNA structure.

Based on previous studies in rice[16, 17], which is a close species to wheat, the GC content is likely to affect the preference of certain tRNAs, thereby, translation, rather than via *in vivo* RNA structure.

To further articulate these possible explanations as suggested, we have added sentences into the discussion section, in lines 487-489, lines 510-511, lines 513-515.

**Rebuttal Figure 6 Gene ontology analysis on the homoeologous pairs with significant difference in mRNA abundance in wheat. A** Venn plot showing the overlap between homoeologous pairs with different TE and that with different mRNA abundance. **B** The 20 gene ontology items that were most significantly enriched for homoelogous pairs with mRNA of A subgenome greater than that of B subgenome (A>B). **C** The 20 gene ontology items that were most significantly enriched for homoelogous pairs with mRNA of A subgenome smaller than that of B subgenome (A<B).

**References**

1.	Kralovicova J, Borovska I, Pengelly R, Lee E, Abaffy P, Sindelka R, Grutzner F, Vorechovsky I: **Restriction of an intron size en route to endothermy.** *Nucleic Acids Res* 2021, **49:**2460-2487.

2.	Hajdin CE, Bellaousov S, Huggins W, Leonard CW, Mathews DH, Weeks KM: **Accurate SHAPE-directed RNA secondary structure modeling, including pseudoknots.** *Proc Natl Acad Sci U S A* 2013, **110:**5498-5503.

3.	Ding Y, Tang Y, Kwok CK, Zhang Y, Bevilacqua PC, Assmann SM: **In vivo genome-wide profiling of RNA secondary structure reveals novel regulatory features.** *Nature* 2014, **505:**696-700.

4.	Bai Y, Dai X, Harrison A, Johnston C, Chen M: **Toward a next-generation atlas of RNA secondary structure.** *Brief Bioinform* 2016, **17:**63-77.

5.	Rouskin S, Zubradt M, Washietl S, Kellis M, Weissman JS: **Genome-wide probing of RNA structure reveals active unfolding of mRNA structures in vivo.** *Nature* 2014, **505:**701-705.

6.	Spitale RC, Flynn RA, Zhang QC, Crisalli P, Lee B, Jung JW, Kuchelmeister HY, Batista PJ, Torre EA, Kool ET, Chang HY: **Structural imprints in vivo decode RNA regulatory mechanisms.** *Nature* 2015, **519:**486-490.

7.	Danecek P, Auton A, Abecasis G, Albers CA, Banks E, DePristo MA, Handsaker RE, Lunter G, Marth GT, Sherry ST, et al: **The variant call format and VCFtools.** *Bioinformatics* 2011, **27:**2156-2158.

8.	Zhou Y, Zhao X, Li Y, Xu J, Bi A, Kang L, Xu D, Chen H, Wang Y, Wang YG, et al: **Triticum population sequencing provides insights into wheat adaptation.** *Nat Genet* 2020, **52:**1412-1422.

9.	Holsinger KE, Weir BS: **Genetics in geographically structured populations: defining, estimating and interpreting F(ST).** *Nat Rev Genet* 2009, **10:**639-650.

10.	Boado RJ, Pardridge WM: **Amplification of gene expression using both 5′- and 3′-untranslated regions of GLUT1 glucose transporter mRNA.** *Molecular Brain Research* 1999, **63:**371-374.

11.	Lytle JR, Yario TA, Steitz JA: **Target mRNAs are repressed as efficiently by microRNA-binding sites in the 5' UTR as in the 3' UTR.** *Proc Natl Acad Sci U S A* 2007, **104:**9667-9672.

12.	dos Reis M, Savva R, Wernisch L: **Solving the riddle of codon usage preferences: a test for translational selection.** *Nucleic Acids Res* 2004, **32:**5036-5044.

13.	Sharp PM, Li WH: **The codon Adaptation Index--a measure of directional synonymous codon usage bias, and its potential applications.** *Nucleic Acids Res* 1987, **15:**1281-1295.

14.	Shabalina SA, Ogurtsov AY, Spiridonov NA: **A periodic pattern of mRNA secondary structure created by the genetic code.** *Nucleic Acids Res* 2006, **34:**2428-2437.

15.	Kertesz M, Wan Y, Mazor E, Rinn JL, Nutter RC, Chang HY, Segal E: **Genome-wide measurement of RNA secondary structure in yeast.** *Nature* 2010, **467:**103-107.

16.	Zhao D, Hamilton JP, Hardigan M, Yin D, He T, Vaillancourt B, Reynoso M, Pauluzzi G, Funkhouser S, Cui Y, et al: **Analysis of Ribosome-Associated mRNAs in Rice Reveals the Importance of Transcript Size and GC Content in Translation.** *G3 (Bethesda)* 2017, **7:**203-219.

17.	Deng H, Cheema J, Zhang H, Woolfenden H, Norris M, Liu Z, Liu Q, Yang X, Yang M, Deng X, et al: **Rice In Vivo RNA Structurome Reveals RNA Secondary Structure Conservation and Divergence in Plants.** *Mol Plant* 2018, **11:**607-622.

18.	Sun L, Fazal FM, Li P, Broughton JP, Lee B, Tang L, Huang W, Kool ET, Chang HY, Zhang QC: **RNA structure maps across mammalian cellular compartments.** *Nat Struct Mol Biol* 2019, **26:**322-330.

19.	Beaudoin JD, Novoa EM, Vejnar CE, Yartseva V, Takacs CM, Kellis M, Giraldez AJ: **Analyses of mRNA structure dynamics identify embryonic gene regulatory programs.** *Nat Struct Mol Biol* 2018, **25:**677-686.

**Second round of review**

**Reviewer 1**

The authors have addressed all my concerns. With the new experiments they performed, the manuscript has greatly improved. I would therefore recommend it for publication in Genome Biology.
